# Study on the Effect and Mechanism of Huaji Jianpi Decoction on Simple Obesity

**DOI:** 10.1155/2022/5494224

**Published:** 2022-04-28

**Authors:** Mengmeng Zhu, Baixia Zhang, Jinghui Zhang, Shaoqin Ge, Meiyun Liu

**Affiliations:** ^1^College of Chinese Medicine, Hebei University, Baoding, Hebei 071000, China; ^2^General Hospital of Xuzhou Mining Group, Xuzhou, Jiangsu 221006, China; ^3^The Center for Reproductive Medicine of Affiliated Hospital of Hebei University, Hebei University, Baoding, Hebei 071000, China

## Abstract

**Background:**

As the major type of obesity in clinical, simple obesity has gained increasing attention in recent years. Depending on the etiology and pathogenesis of simple obesity and combined with clinical practice experience, Huaji Jianpi decoction (HJJPD) was established to invigorate the spleen and eliminate dampness; however, the underlying molecular mechanism is yet unclear.

**Materials and Methods:**

A simple obesity mouse model was established by feeding a high-fat diet to the animals, and the related indexes were analyzed. The mice were divided into the normal, positive control (orlistat), and HJJPD high-dose, medium-dose, and low-dose groups. After 6 weeks of administration, the curative effect of HJJPD was observed. Simple obesity is associated with leptin resistance. The leptin signal transduction pathways mainly include the JAK2-STAT3, AMPK-ACC, LepRb-IRS-PI3K-PDE3B-cAMP, and LepRb-SHP2-MAPKs (ERK1/2) pathways. Therefore, the networks of HJJPD acting on these four pathway-related targets were constructed using the network pharmacology method, and the key nodes were identified.

**Results:**

After 6 weeks of drug intervention, we found a good therapeutic effect of HJJPD on simple obesity in the mouse model. The biological network analysis showed that HJJPD plays a role in treating leptin resistance in simple obesity by acting on multiple targets in the JAK2-STAT3 pathway via various components. Also, HJJPD can improve leptin resistance in mice by enhancing the binding force of LEP and LEPRB and activating the LEP-mediated JAK2-STAT3 signaling pathway.

**Conclusion:**

In this study, animal experiments, network pharmacology, and molecular biology were combined to establish a mouse model of simple obesity, confirm the role of HJJPD in the treatment of simple obesity, and preliminarily reveal the related mechanism. Relevant research results will provide a basis for the treatment of simple obesity and the drug discovery.

## 1. Introduction

During the past decades, obesity has become a global public health problem. It was reported that obesity was not only a critical risk factor for metabolic diseases but also associated with cancer and reproductive system diseases significantly [1, 2]. Obesity can be divided into two types clinically according to the etiology: simple and secondary. Simple obesity is the most common type of clinical obesity that accounts for more than 95% of the total cases. The clinical manifestations of simple obesity are no obvious changes in the functions of the nervous and endocrine systems but impede the glucose and lipid metabolism regulation in vivo. Since it has no obvious primary disease, simple obesity has become a challenging topic in clinical research.

Currently, the clinical treatment of obesity includes lifestyle changes, western medicine, surgery, and traditional Chinese medicine (TCM). Obese patients do not easily accept western medicine due to efficiency and safety concerns [3, 4]. Gastrointestinal surgery requires strict indications, is costly, and may lead to postoperative complications [5]. TCM has a long history of contributing to the discovery of new drugs and the treatment of complex diseases [6–8]. In the treatment of obesity, TCM has fewer side effects and lasting efficacy, especially in reducing the number of fat cells, improving the quality of life, and avoiding severe complications [9, 10]. Therefore, a large number of patients with simple obesity choose TCM to lose weight.

According to the theory of TCM, simple obesity shows dysfunction of the spleen in transport and phlegm turbidity; the basic treatment method of strengthening the spleen and clearing dampness is summarized previously [11]. Depending on the etiology and pathogenesis of simple obesity combined with clinical practice experience, “Huaji Jianpi decoction (HJJPD)” was established based on Zhizhu pills, Liujunzi decoction, and the principle of invigorating spleen to eliminate dampness. This recipe consists of roasted *Aurantii fructus iimmaturus* (15 g), roasted *Atractylodis rhizoma* (15 g), *Poria* (30 g), *Pinelliae rhizoma* (10 g), *Citri reticulatae pericarpium* (10 g), *Nelumbinis folium* (10 g), *Astragali radix* (30 g), *Chuanxiong rhizoma* (10 g), roasted *Coicis semen* (30 g), and *Alismatis rhizoma* (10 g).

A previous study has shown that simple obesity is associated with leptin resistance [12, 13]. The abnormal leptin signal transduction is the leading mechanism underlying leptin resistance [14]. The leptin signal transduction pathways have been discovered recently, including JAK2-STAT3, AMPK-ACC, LepRb-IRS-PI3K-PDE3B-cAMP, and LepRb-SHP2-MAPKs (ERK1/2) [15–17]. In the present study, the male mouse model of simple obesity was constructed to verify the efficacy of HJJPD for the treatment of the condition. A biological network of HJJPD acting on the targets related to the four pathways was constructed by the network pharmacology method. The role of HJJPD in treating simple obesity was explored, and the key nodes were identified.

## 2. Materials and Methods

### 2.1. Animals and Reagents

Male C57BL/6N mice (3-week old, SPF grade) were purchased from Beijing Vital River Laboratory Animal Technology Co., Ltd. (Beijing, China). Insulin injections were purchased from Jiangsu Wanbang Biochemical and Pharmaceutical Group Co., Ltd. (Jiangsu, China). Glucose injection was purchased from Shijiazhuang No. 4 Pharmaceutical Co., Ltd. (Shijiazhuang, China). Pentobarbital sodium, Bouin's fixed fluid, xylene, and hematoxylin-eosin (HE) dye were bought from Tianjin Fuchen Chemical Reagent Co. Ltd. (Tianjin, China). Mouse insulin (INS) enzyme-linked immunosorbent assay (ELISA), free fatty acid (FFA) ELISA, leptin ELISA, adiponectin ELISA, and interleukin 6 (IL-6) ELISA kits were obtained from Beijing Koren Technology Co., Ltd. (Beijing, China). TriPure reagent for total RNA extraction was purchased from Shijiazhuang Huawokorui Biotechnology Co., Ltd. (Shijiazhuang, China).

### 2.2. Drug Preparation

The medicinal materials of HJJPD (600 g) were soaked in water for 30 min, boiled with high fire, decocted gently for 1 h, and filtered, and the filtrate was retained. Then, water was added and decocted for 40 min, followed by filtration. The filtrates were pooled and concentrated to a volume of 120 mL (5 g/mL). High, medium, and low doses of the drug (4.55, 3.03, and 2.02 g/mL, respectively) were prepared with distilled water and stored at 4°C. Orlistat capsule (0.45 g) was ground into powder to prepare a concentration of 0.006825 g/mL and stored at 4°C.

### 2.3. Establishment of a Simple Obesity Mice Model

A total of 100 male C57BL/6N mice were randomly divided into normal (*n* = 20) and model (*n* = 80) groups. After adaptive feeding for one week, the model group was fed a high-fat diet, and the normal group was fed a maintenance diet. The model was constructed for 10 weeks. This study was approved by the animal ethics committee of Hebei University, China. All protocols were carried out in accordance with the Guidelines for the Care and Use of Laboratory Animals in China.

### 2.4. Methods for Determination of Obesity-Related Indexes

The physiological state of the mice was observed during the modeling period. The animals in the two groups were weighed weekly, and the growth curve was drawn. Body length and waist circumference were measured after modeling. Lee's index in each group was calculated according to the following formula:

(1) $$\begin{array}{c} \text{Lee's index}=\frac{{\text{body weight}}^{1/3}\left(\text{g}\right)}{\text{body length}\left(\text{cm}\right)}\times 103. \end{array}$$

Six weeks after administration, the mice were subjected to a glucose tolerance test (GTT) and insulin tolerance test (ITT). The animals were anesthetized with 1% pentobarbital sodium, and the total fat volume in each group was detected using micro-CT. The eyeballs of the mice were removed for blood collection. The expression levels of aspartate aminotransferase (AST), alanine aminotransferase (ALT), gamma-glutamyl transferase (GGT), glucose (GLU), total cholesterol (TC), triglyceride (TG), high-density lipoprotein (HDL), low-density lipoprotein (LDL), and very-low-density lipoprotein (VLDL) in serum were detected using the Automated Bioanalyzer. The morphological and structural differences in the liver and adipose tissue were observed. The tissues were fixed with Bouin's fixative solution and dehydrated. After cooling and embedding in paraffin, 5 *μ*m thick sections were successively cut and dewaxed by drying. Subsequently, the slices were soaked in hematoxylin dye and dehydrated. After restaining in eosin dye for 8 min, the slices were dehydrated with conventional gradient alcohol, sealed, labeled, and observed under an optical microscope.

### 2.5. Effects of HJJPD on Glucose Metabolism, Lipid Metabolism, and Liver and Adipose Histology of Mice

A total of 10 mice from the normal group were randomly selected to form the normal control group, and the simple obese mice in the model group were randomly divided into the model, positive control, and HJJPD high-dose, medium-dose, and low-dose treatment groups (*n* = 10 mice/group). Gavage was performed every morning (9:00): 0.10 g/kg/d in the positive control group and 68.18, 45.45, and 30.30 g/kg/d in the high-, medium-, and low-dose HJJPD groups, respectively. The normal and blank model groups were given equivalent volumes of distilled water by gavage for 6 weeks. The drug was maintained in a 37°C water bath for 20 min before use. The administration dose was adjusted according to the weekly bodyweight change of the mice in each group. The animals in the remaining groups were still fed a high-fat diet, except for the normal group during administration. After 6 weeks of drug intervention, the bodyweight, body length, and waist circumference were measured, and Lee's index was calculated. The obesity-related indicators were determined using the method described in this section. The levels of INS and FFA in each group were detected by ELISA kits.

### 2.6. Data for Building Biological Networks

The components and targets of HJJPD were collected from the TCMSP database (https://tcmspw.com/tcmsp.php). The active compounds were screened according to the conditions of oral bioavailability (OB ≥ 30%) and drug-likeness (DL ≥ 0.18). The full names of the action targets were converted into short names by the Uniprot database. Data on proteins associated with the JAK2-STAT3, AMPK-ACC, LepRb-SHP2-MAPKs (ERK1/2), and LepRb-IRS-PI3K-PDE3B-cAMP pathways were obtained from the literature [15–17]. Protein interaction data were obtained from the STRING database, and the proteins with confidence ≥0.7 were selected as the component and the interaction targets of the JAK2-STAT3 pathway.

### 2.7. Construction and Analysis of Biological Networks

Cytoscape 3.7.1 was used to construct the biological network of HJJPD acting on the pathway-related targets, showing an interaction correlation between the components and proteins. The CytoNCA in Cytoscape3.7.1 was used to analyze the topological structure of the network. Nodes with betweenness and closeness greater than the median were regarded as the key nodes. The molecules targeting the key nodes were the effective components of HJJPD acting on the pathway. MCODE in Cytoscape was used for module analysis of the biological network, and David 6.8 was used for gene ontology (GO) and Kyoto Encyclopedia of Genes and Genomes (KEGG) pathway enrichment analysis of the identified modules.

### 2.8. Effects of HJJPD on Adipokine and JAK2-STAT3 Signaling Pathway in Mice with Simple Obesity

After 6 weeks of treatment, the mice were anesthetized with sodium pentobarbital (1%), and the serum and tissue samples were collected. The levels of LEP, ADP, and IL-6 in the serum of each group were detected by ELISA kits. The mRNA expression levels of LEPRB, SOCS-3, JAK2, and STAT3 in the liver and adipose tissue were detected by RT-qPCR to verify the effect of HJJPD on the JAK2-STAT3 pathway.

### 2.9. Statistical Analysis

The experimental data were analyzed using SPSS 22.0. The measurement data were represented as $\bar{x}\pm s$. The intuitive analysis method and independent sample *t*-test were used for statistical analysis. One-way analysis of variance (ANOVA) was used for comparison between multiple groups of samples. *P* < 0.05 indicated statistical significance.

## 3. Results

### 3.1. Construction and Evaluation of a Simple Obesity Male Mouse Model

To evaluate the simple obesity male mouse model, we performed a general index analysis, histomorphological analysis, and serological tests.

#### 3.1.1. General Index Analysis of Mice

**Figure 1:**
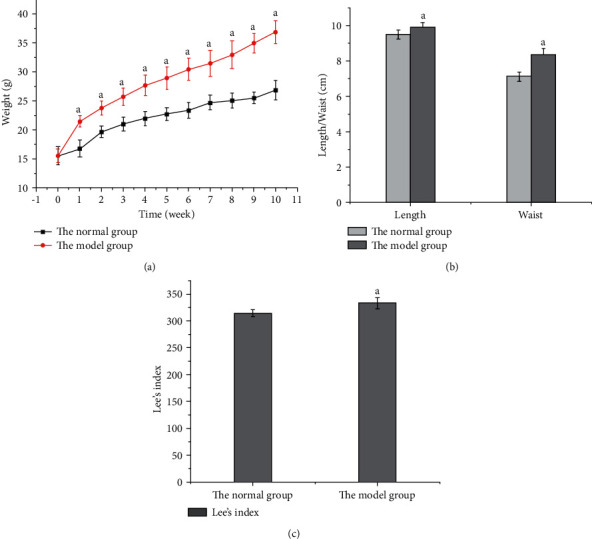
Comparison of bodyweight, body length, waist circumference, and Lee's index between the model and normal groups. (a) Bodyweight growth curves of the two groups of mice during modeling. (b) Comparison of body length and waist circumference. (c) Comparison of Lee's index.

The commonly used evaluation indexes of obesity include bodyweight and body fat; bodyweight is an intuitive index, and body fat is the most accurate index. After feeding a high-fat diet for 10 weeks, the bodyweight of the model group was 37.19% higher than that of the normal group (*P* < 0.05, Figure 1(a)). Out of 80 mice, 71 were successfully modeled in the model group, with a success rate of 88.75%. The body length, waist circumference, and Lee's index of the model group were higher than those of the normal group (*P* < 0.05, Figure 1(b)).

**Figure 2:**
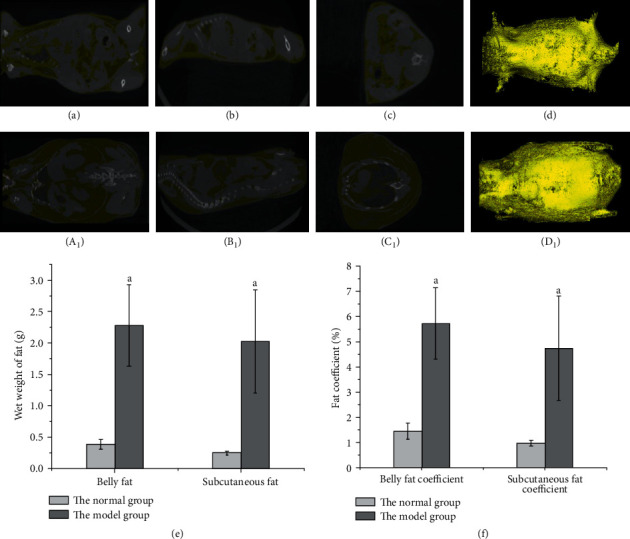
Comparison of fat distribution, wet fat weight, and fat coefficient in the two groups of mice. *Note*. Yellow represents fat; white represents other tissues. (d) Stereogram of fat distribution in normal mice synthesized by (a), (b), and (c). (a) Upper section, (b) forward section, and (c) lateral section. (d_1_) Stereogram of fat distribution of model group mice synthesized by (a_1_), (b_1_), and (c_1_). (a_1_) Upper section, (b_1_) forward section, and (c_1_) lateral section. (e) The wet weight of the abdominal cavity and subcutaneous fat. (f) Abdominal and subcutaneous fat coefficients.

The micro-CT scanning revealed that the distribution of fat in mice could be analyzed intuitively, and the total fat volume was obtained. The comparison between the two groups showed that the total fat volume, the distribution of the subcutaneous, abdominal, and visceral fat, the wet weight, and the coefficient of the abdominal cavity and subcutaneous adipose tissue were significantly higher in the model group than in the normal group (Figures 2(a)–2(f); *P* < 0.05). The body fat content of obese mice can be analyzed comprehensively by combining fat distribution, fat volume, and wet fat weight.

#### 3.1.2. Serological Tests

**Figure 3:**
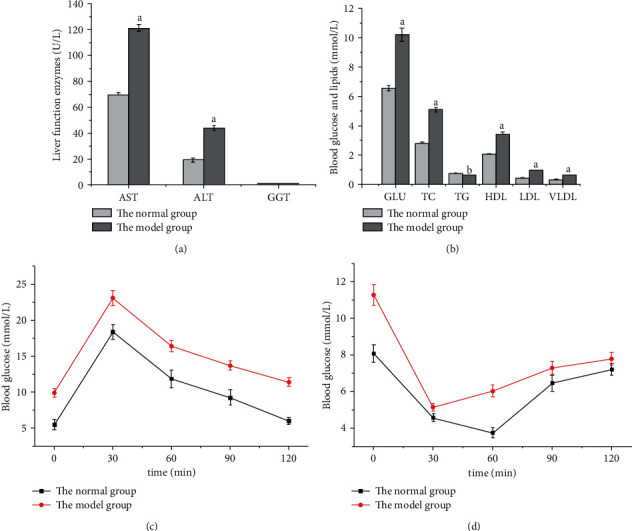
Comparison of liver enzyme tests, GTT, and ITT between model and normal groups. (a) Comparison of liver function enzyme levels. (b) Comparison of blood GLU and lipid levels. (c) Blood GLU changes in GTT. (d) Blood GLU changes in ITT.

Previous studies have shown that obesity can alter blood GLU, lipid, liver function indexes, and endocrine hormone levels [18]. Therefore, in addition to bodyweight, Lee's index, and body fat, the levels of AST, ALT, GGT, GLU, TC, TG, HDL, LDL, and VLDL in serum were also critical indicators to evaluate the success of the animal model of simple obesity. As shown in Figure 3(a), AST and ALT levels in the model group were higher than those in the normal group, but the GGT level was similar in the two groups. The GLU, TC, HDL, LDL, and VLDL levels were higher, and the TG level was lower in the model group than in the normal group (Figure 3(b); *P* < 0.05).

The combined determination of lipid content with GTT and ITT could better analyze the phenomenon of glucose and lipid metabolism disorder in obese mice than when used alone. The GTT results showed that the blood glucose of the two groups was maximal at 30 min and then began to decrease uniformly, but the blood GLU of the model group was higher than that of the normal group at each detection time point (Figure 3(c); *P* < 0.05), indicating that the mice in the model group had abnormal GLU tolerance. ITT results showed that the blood GLU of mice in the normal group decreased to the lowest level at 60 min, while that in the model group decreased to the lowest level at 30 min, and the levels in the model group were higher than those in the normal group at each detection time point (Figure 3(d); *P* < 0.05).

#### 3.1.3. Histomorphological Analysis

**Figure 4:**
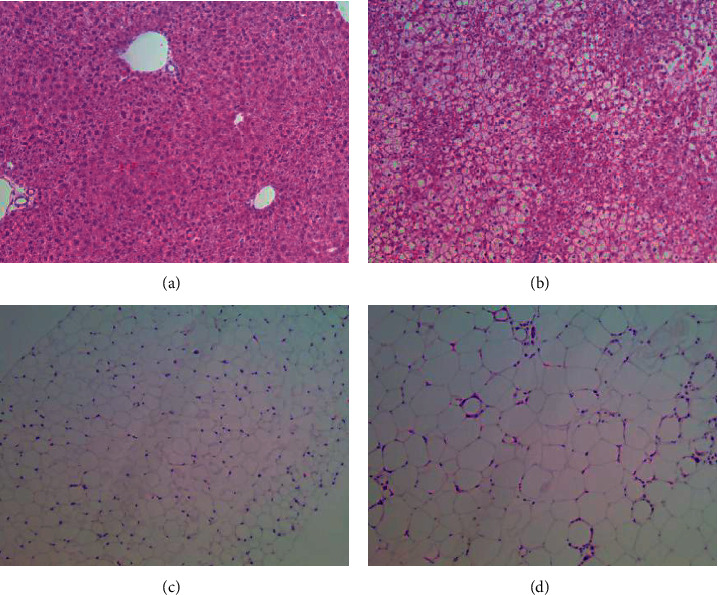
Morphology and structure of the liver and adipose tissue in the two groups. (a) Liver structure in the normal group (HE, ×100). (b) Liver structure in the model group (HE, ×100). (c) Adipose tissue structure in the normal group (HE, ×100). (d) Adipose tissue structure in the model group (HE ×100).

The histological features of obesity are fat cell enlargement and liver cell steatosis; thus, we carried out a histomorphological analysis. The comparison of liver and adipose tissue morphology between the two groups is shown in Figure 4. The liver structure of the mice in the normal group was normal with an orderly arrangement of liver cells, while the liver structure of the model group mice was disordered with a large number of lipid droplet vacuoles of different sizes in the cytoplasm and steatosis of liver cells (Figures 4(a) and 4(b)). The adipose tissue structure of the mice in the normal group was normal, with uniform size and a small volume of adipose cells, while the cells in the model group had an irregular morphology and significantly increased volume, accompanied by infiltration of inflammatory cells (Figures 4(c) and 4(d)).

### 3.2. Effect of HJJPD on Simple Obese Male Mice

#### 3.2.1. Effect of HJJPD on the General Index Analysis of Simple Obese Male Mice

**Figure 5:**
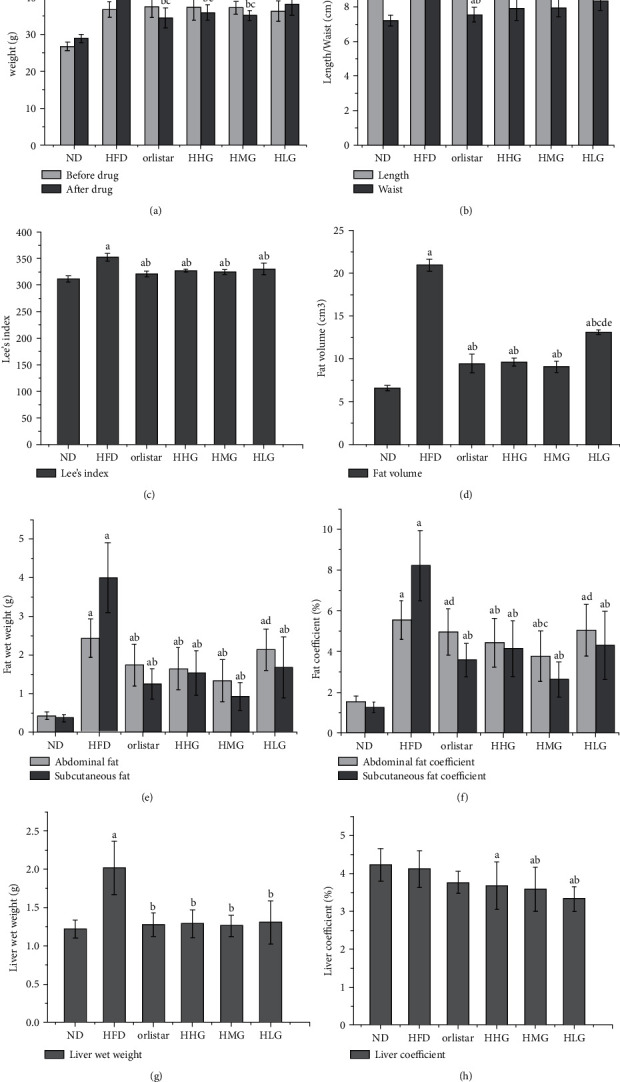
Effects of HJJPD on bodyweight, adipose tissue, and liver tissue in mice. (a) Changes in bodyweight of mice before and after administration. (b) Effects of HJJPD on body length and waist circumference in mice. (c) Effect of HJJPD on Lee's index in mice. (d) Total fat volume in mice of each group after administration. (e) Effect on wet weight of fat. (f) Effect on fat coefficient. (g) Effect on the wet weight of the liver. (h) Effect on liver coefficient.

In order to evaluate the therapeutic effect of HJJPD on simple obesity, we analyzed the general body indexes of mice, including bodyweight, Lee's index, and adipose coefficient. Lee's index was more sensitive to obesity in mice and highly correlated with bodyweight and fat volume compared to the body mass index. After 10 weeks of modeling, the bodyweight of the other 5 groups did not differ significantly (*P* > 0.05), except for the normal group, but the bodyweight was higher than that of the normal group (Figure 5(a)). After 6 weeks of administration, the bodyweight of mice in all the administered groups decreased markedly compared to the model group (*P* < 0.05), while no difference was observed between the positive control, high-dose, and medium-dose groups (*P* > 0.05), but the bodyweight of the low-dose group was higher than that of the other three groups (*P* < 0.05). The effects of HJJPD on body length, waist circumference, and Lee's index of mice are shown in Figures 5(b) and 5(c). Except for the low-dose group, the body length of mice in each administered group was smaller than that in the model group (*P* < 0.05). Compared to the model group, the waist circumference and Lee's index of high-dose, medium-dose, and low-dose groups were decreased (*P* < 0.05), but no difference was observed among all groups (*P* > 0.05).

Compared to the model group, the total fat volume of mice in each administered group decreased (*P* < 0.05), and no difference was detected between the other administered groups, except for the low-dose group (Figure 5(d); *P* > 0.05). The wet weight and coefficient of fat in each administered group decreased to varying degrees (*P* < 0.05), and no difference was observed in the wet weight of abdominal fat and subcutaneous fat between the positive control and the high-dose, medium-dose groups (Figures 5(e) and 5(f); *P* > 0.05). Compared to the other administered groups, the improvement in the wet weight and coefficient of adipose tissue in the low-dose group was lower. The wet weight of the liver in each administered group was decreased (*P* < 0.05), and no difference was detected among all the administered groups (*P* > 0.05). The liver coefficients of the high-dose, medium-dose, and low-dose groups were lower than those of the normal group (*P* < 0.05), and those of the medium-dose and low-dose groups were lower than those of the model group (Figures 5(g) and 5(h); *P* < 0.05).

#### 3.2.2. Effect of HJJPD on Liver Tissue and Adipose Tissue of Simple Obese Male Mice

**Figure 6:**
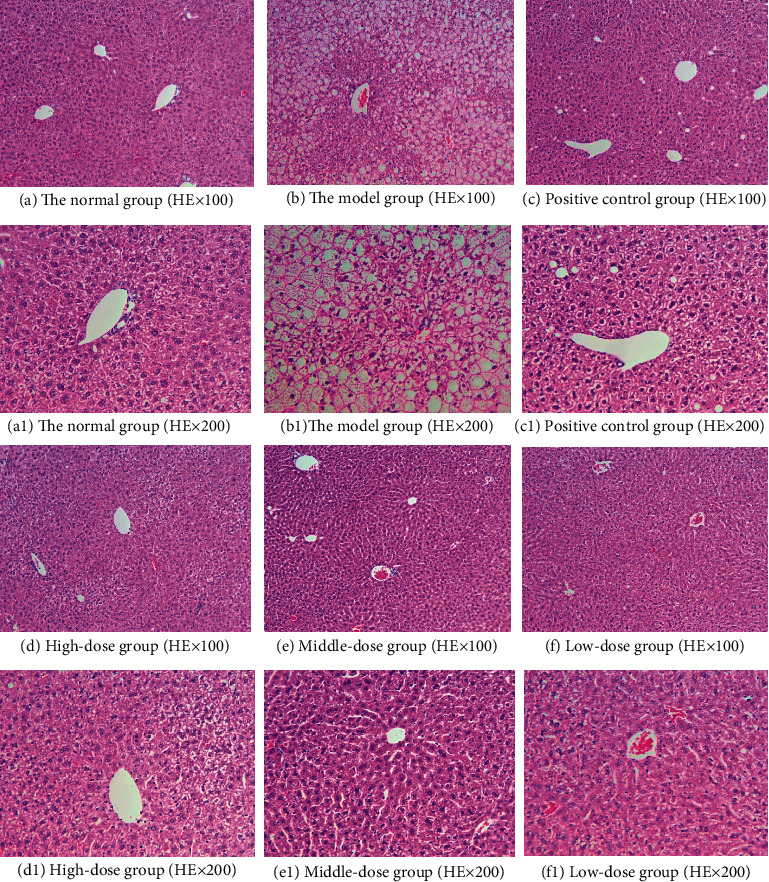
Liver tissue structure of mice in each group after administration.

In order to study the effect of HJJPD on adipose cells of mice with simple obesity, we evaluated the morphology of the liver and adipose tissue. As shown in Figure 6, the liver structure of the normal group was normal without inflammatory cell infiltration. The liver structure of the model group was disorganized, and the liver cells showed steatosis, increased volume and infiltration by inflammatory cells, and a large number of lipid vacuoles of different sizes in the cytoplasm. Compared to the model group, the arrangement of liver cells in the high-dose, medium-dose, and low-dose groups was uniform and orderly arranged, and no lipid vacuoles were detected in the cytoplasm, which significantly improved the enlargement of cell volume and steatosis, and a small number of lipid vacuoles was detected in the positive control group.

**Figure 7:**
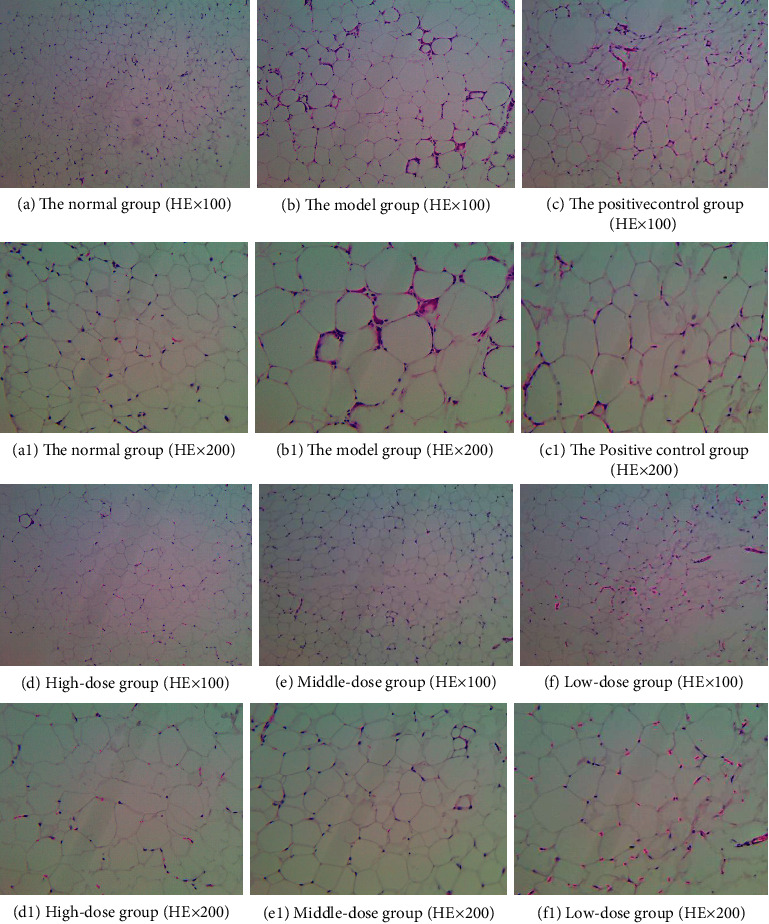
Morphological structure of adipose tissue in each group after administration.

As shown in Figure 7, the morphology and structure of adipose tissue of mice in each group were observed under a light microscope. The adipose tissue structure of mice in the normal group was normal, and the adipose cells were small, uniform in size, and complete in morphology. Compared with the normal group, the volume of adipocytes in the model group was significantly increased, and a large number of inflammatory cells were infiltrated. The cell morphology was irregular, and the cells tended to fuse and merge with each other, leading to an increase in the volume and a decrease in the number of cells. After drug intervention, compared with the model group, the volume of adipocytes in the high-dose, medium-dose, and low-dose groups was significantly reduced, and the number of adipocytes in the observed unit field of vision was increased. There was basically no inflammatory cell infiltration in high and medium-dose groups, but the effect of the low-dose group was poor. The positive control group had no obvious improvement on adipocytes of mice.

### 3.3. HJJPD Plays a Role in the Treatment of Simple Obesity through JAK2-STAT3 Pathway

#### 3.3.1. Biological Network of HJJPD Acts on the Proteins Related to the JAK2-STAT3 Pathway

**Figure 8:**
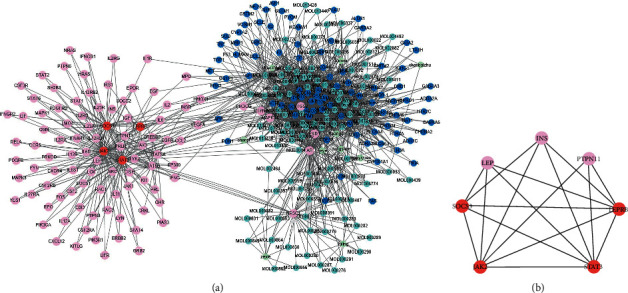
Biological network of HJJPD acts on the proteins related to the JAK2-STAT3 pathway.

After screening, 115 components meeting OB ≥ 30% and DL ≥ 0.18 (Supplementary Table S1) were obtained, and a total of 169 acting targets were obtained (Supplementary Table S2). The biological networks of HJJPD acting on the JAK2-STAT3 pathway, AMPK-ACC pathway, LepRb-IRS-PI3K-PDE3B-cAMP pathway, and LepRb-SHP2-MAPKs (ERK1/2) pathway-related targets were constructed, respectively (Supplementary S3). The network analysis revealed that HJJPD could act on more JAK2-STAT3 pathway-related proteins than the other three pathways. Therefore, compared to other signaling pathways, HJJPD is more likely to play a role in the treatment of simple obesity by acting on the JAK2-STAT3 pathway. The network of HJJPD acts on the proteins related to the JAK2-STAT3 pathway (Figure 8(a)); green nodes represent herbs, light blue nodes represent components, dark blue nodes represent the targets of the components, red nodes represent related proteins of the JAK2-STAT3 pathway, and pink nodes represent the targets that interact with proteins associated with the JAK2-STAT3 pathway.

#### 3.3.2. Key Components of the JAK2-STAT3 Pathway Acting via HJJPD

The topological analysis identified the key component of HJJPD acting on the JAK2-STAT3 pathway: cavidine, 7-methoxy-2-methyl isoflavone, 7-O-methylisomucronulatol, beta-sitosterol, kaempferol, and quercetin (Table 1).

#### 3.3.3. Functional Modules of the Network of Proteins Associated with the JAK2-STAT3 Pathway Acted by HJJPD

A total of five functional modules were obtained through module analysis (Table 2). Modules 1, 2, 4, and 5 encompass a large number of components and a small number of proteins that affect the JAK2-STAT3 pathway. SOCS3, JAK2, STAT3, and LEPRB in module 3 (Figure 8(b)) are components of the JAK2-STAT3 pathway. Reportedly, INS and LEP are closely related to obesity [19, 20], while PTPN11 was identified in the studies of histiocytic sarcoma and Noonan syndrome [21].

The enrichment analysis showed that the proteins in module 3 are mainly involved in three biological processes (Table 3). Except for the JAK2-STAT3 signaling pathway, the adipocytokine signaling pathway and IL-6-type cytokine-signal-transduction are also closely related to obesity [22–25].

#### 3.3.4. Effect of HJJPD on SOCS-3, JAK2, LepRB, LEP, IL-6, and STAT3 Related to the JAK2-STAT3 Pathway in Mouse

Network analysis showed that SOCS-3, JAK2, LepRB, LEP, IL-6, and STAT3 are critical targets. Therefore, we studied the changes in the expression levels of the above targets to verify whether HJJPD plays a role in the treatment of simple obesity through the JAK2-STAT3 pathway (Table 4). Compared to the normal group, the gene expression levels of LepRB, JAK2, and STAT3 in the liver of the model group were decreased, while the expression level of SOCS-3 was significantly increased. Moreover, the expression of LepRb, JAK2, and STAT3 in the low-dose group and the expression of JAK2 in the positive control group did not differ significantly, but that of LepRb, JAK2, and STAT3 in all the treatment groups was increased markedly. The expression of SOCS-3 in the positive control group did not differ significantly different, but that in the other groups decreased to varying degrees.

Gene expression levels of LepRB, JAK2, and STAT3 in the adipose tissue of mice in the model group were decreased compared to those in the normal group (*P* < 0.05), while that of SOCS-3 was significantly increased (*P* < 0.05, Table 5). Compared to the model group, the expression levels of LepRb and JAK2 in the positive control group and LepRb and STAT3 in the low-dose group did not differ significantly (*P* > 0.05); however, the expression levels of LepRb, JAK2, and STAT3 in the other treatment groups were increased to varying degrees (*P* < 0.05). Compared to the model group, the expression level of SOCS-3 in the other administrative groups decreased to varying degrees (*P* < 0.05), while that of SOCS-3 showed no difference in the positive control group (*P* > 0.05).

**Figure 9:**
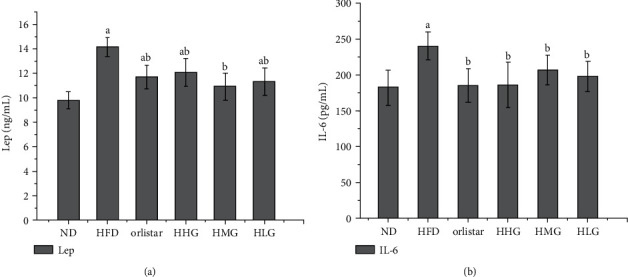
Lep and IL-6 levels of mice in each group.

After 6 weeks of administration, the Lep level in all the groups, except the medium-dose group, was higher than that in the normal group (*P* < 0.05). However, the Lep level of mice in each administered group improved to different degrees compared to that in the model group (*P* < 0.05), but no difference was observed among all the administered groups (Figure 9(a)). The level of IL-6 in the model group was significantly higher than that in the normal group (*P* < 0.05). After 6 weeks of drug intervention, the level of IL-6 in each administered group was lower than that in the model group (*P* < 0.05; Figure 9(b)), and no difference was detected among the administered groups.

## 4. Discussion

An appropriate animal model is essential to reveal the mechanism of obesity and drug action. Simple obesity refers to no other induced disease, only because the intake of energy is more than the consumption. Therefore, to mimic the state of simple obesity, the mouse model of simple obesity was established in this study by feeding a high-fat diet. After 1 week of feeding, the bodyweight of the two groups showed significant changes. After the second week, the bodyweight of the model group increased significantly and continued to the end of the experiment. Compared to the normal group, the average increase was 37.2%. The fat distribution, the total fat volume, the wet weight, and the coefficient of adipose tissue were higher in the model group than that in the normal group. ALT and AST were mainly distributed in liver cells, and the degree of increase is proportional to the degree of damage to the liver cells [26]. Elevated ALT and AST indicate liver damage, of which ALT is rather sensitive. A two-fold increase in serum ALT indicates necrosis in 1% of liver cells. The results showed that the serum AST and ALT levels in the model group were higher than those in the normal group, indicating mild liver injury, while the GGT level showed no significant change, indicating no serious liver injury. The serum GLU, TC, HDL, LDL, and VLDL levels were increased in the model group and positively correlated with bodyweight. The altered GTT blood GLU showed that the fasting blood GLU in the model group was significantly higher than that in the normal group, and the mice in the model group had abnormal GLU tolerance. The changes in ITT blood GLU showed that the fasting blood GLU of the model group was higher than that of the normal group, suggesting a phenomenon of insulin resistance. Although GTT and ITT showed IGT and IR in the model group, fasting GLU levels were within the normal range. In this study, a simple obesity mouse model was established using a high-fat diet, and the model was evaluated from three aspects: general condition, serological, and histomorphological analyses. The evaluation results showed that the mice in the model group fulfilled the basic characteristics of simple obesity and could be used as an animal model for research on the condition. The model is simple to operate and has a short modeling period. It is stable, which lays a foundation for the follow-up study on the efficacy and mechanism of HJJPD in the treatment of simple obesity mice.

After HJJPD intervention, compared to the model group, the bodyweight and Lee's index of mice in the high- and medium-dose groups and the positive control group were significantly reduced, indicating that these three groups could control the weight growth of mice and reduce the bodyweight to achieve the effect of weight loss. The bodyweight and Lee's index of the low-dose group decreased, but the effect of bodyweight control was not obvious. The increase in subcutaneous fat may be one of the factors leading to high leptin levels in obese mice [27]. HJJPD reduces the fat volume, the wet weight of the abdominal cavity, and subcutaneous adipose tissue in mice to reduce weight loss, lipid, and leptin levels. HJJPD also reduces the wet weight of the liver of mice, treats the severe steatosis of the liver cells of obese mice, reduces the heterotopic deposition of fat in the liver, relieves liver function injury, and regulates the abnormal liver lipid metabolism. The analysis of pathological sections showed a large number of lipid vacuoles in the liver tissue of mice in the model group, the liver cells were destroyed, and the lipid droplets accumulated in large quantities. After treatment with HJJPD, the liver morphology of mice was similar to that of the normal group without the accumulation of lipid droplets, and fewer fat droplets were detected in the hepatocytes of mice in the positive control group. In addition, the histological characteristic of obesity was an increase in adipocyte volume. The analysis of pathological sections showed that the model group mice had an increase in adipocyte volume and uneven size accompanied by obvious infiltration of inflammatory cells. The morphological structure of the adipocyte tissue of mice in each administered group had improved to varying degrees. High- and medium-dose HJJPD groups exhibited reduced size of adipocytes, improved cell morphology, and reduced infiltration of inflammatory cells. Taken together, HJJPD reduces the bodyweight and body fat of obese mice compared to the positive control group, no shit thin pond shapeless adverse reactions were observed, the morphology of the liver and adipose tissue was restored, fatty degeneration of hepatic cells was prevented, the liver function was improved, the volume size of fat cells was decreased, and the inflammatory cell infiltration was improved. In addition, the study showed that Zhizhu pills and Xiangsha Liujunzi decoction have a higher safety in the treatment of spleen deficiency and qi stagnation. Blood, urine, stool routine, and liver and kidney function of the patient were normal, and no serious adverse reactions occurred [28]. Thus, it could be inferred that HJJPD has good efficacy and safety in the treatment of simple obesity.

Topological analysis and modular analysis revealed the role of HJJPD in the treatment of simple obesity by acting on SOCS-3, JAK2, LepRB, LEP, IL-6, and STAT3 and influencing the JAK2-STAT3 pathway. The above key targets are also involved in the adipocytokine signaling pathway and IL-6-type cytokine-signal-transduction. Obesity-induced leptin resistance leads to abnormal leptin-mediated signaling pathways. Leptin exerts a wide range of physiological functions only after binding to its receptors and passing through the corresponding signaling pathways. The altered levels of leptin in animal models of obesity may reflect the mechanism underlying obesity. Previous studies have shown that the leptin-mediated JAK2-STAT3 signaling pathway is related to obesity. In this study, the expression levels of key genes LepRb, SOCS-3, JAK2, and STAT3 in the JAK2-STAT3 signaling pathway in liver and adipose tissue of mice in each group were analyzed to explore the putative mechanism of HJJPD in treating leptin resistance. The expression levels of LEPRB, SOCS-3, JAK2, and STAT3, and the key targets in the JAK2-STAT3 signaling pathway in liver and adipose tissue of mice in each group, were analyzed to verify the mechanism of HJJPD in the treatment of simple obesity. The results showed that the expression levels of LEPRB, JAK2, and STAT3 in the model group were significantly lower than those in the normal group, while the level of SOCS-3 was significantly higher than that in the normal group. After intragastric administration, compared to the model group, the gene expression levels of LEPRB, JAK2, and STAT3 in each administered group were increased to varying degrees, and that of SOCS-3 was significantly decreased. The analysis of the expression levels of JAK2 and STAT3 genes upstream of LEPRB and downstream of the JAK2-STAT3 signaling pathway and the expression of the negative regulator SOCS-3 confirmed that HJJPD activates the LEP-mediated JAK2-STAT3 signaling pathway in the liver and adipose tissues. The LEP level of mice in each administered group was lower than that of the model group, indicating that HJJPD can improve leptin sensitivity and hyperleptinemia in mice. The IL-6 levels of mice in each administration group decreased to different degrees, indicating that HJJPD alleviates chronic inflammatory responses in mice. In conclusion, this study confirmed that HJJPD improves hyperleptinemia and relieves chronic inflammation in mice by regulating the level of adipokine. The stimulation of the LEP-mediated JAK2-STAT3 signaling pathway in liver and adipose tissue improves leptin resistance of obese mice, thus playing a role in the treatment of simple obesity.

## 5. Conclusions

In this study, a simple obesity model was established successfully, and the effect of HJJPD in treating simple obesity was clarified by animal experiments and molecular biology. In order to explore the mechanism of action of HJJPD in treating simple obesity, the biological network of HJJPD acting on leptin-related signaling pathway was constructed by the network pharmacology method, and the role of HJJPD in treating simple obesity through the JAK2-STAT3 signaling pathway was substantiated. We found that HJJPD reduces hyperleptinemia, alleviates chronic inflammatory response, improves leptin resistance of obese mice, and plays a role in treating simple obesity by activating the LEP-mediated JAK2-STAT3 signaling pathway in liver and adipose tissue. Furthermore, HJJPD has the characteristics of low side effects, lasting therapeutic effect, and can adjust the patient's constitution in the treatment of simple obesity mice. The molecular mechanism of HJJPD in the treatment of simple obesity and the basic treatment method of “strengthening spleen and clearing damp” can provide novel ideas for the clinical treatment of obesity.

## Figures and Tables

**Table 1:** Key component of the JAK2-STAT3 pathway effectuated by HJJPD.

Name	Betweenness centrality	Closeness centrality
Cavidine	0.01991412	0.41838134
7-Methoxy-2-methyl isoflavone	0.02267325	0.41838134
7-O-methylisomucronulatol	0.01940242	0.42068966
Beta-sitosterol	0.04244781	0.43079096
Kaempferol	0.05116754	0.43079096
Quercetin	0.10634613	0.44721408

**Table 2:** Functional modules of the biological network of proteins associated with the JAK2-STAT3 pathway acted by HJJPD.

Module	Score	Nodes	Edges	Node IDs
1	7.25	17	58	PIM1, PRSS1, MOL007207, CA2, MOL007206, CCNA2, MOL000354, MOL007210, MOL007213, COX1, MOL000392, MOL000006, MOL000380, HSP90, MOL000371, MOL007879, and NCOA2
2	6.667	13	40	PPARG, MOL002670, MOL003896, MOL000422, F2, CD26, Cdk2, ESR2, GSK3B, MOL000378, iNOS, MOL000358, and MOL000449
3	4.8	6	15	JAK2, PTPN11, INS, STAT3, LEPRB, and LEP
4	4.5	13	27	Chk1, MOL005321, MOL007059, ACHE, ADRB2, MOL007218, MOL005100, SCN5A, MOL000296, MOL000519, MOL013277, MOL001803, and MOL005828
5	3	7	9	Chrm3, MOL001941, GABRA1, MOL000049, MOL013437, chaozhishi, and CHRM1

**Table 3:** Biological processes in module 3 (*P* < 0.05).

Term	Count	*P* value	Genes
Adipocytokine signaling pathway	4	8.67*E*−06	LEP, JAK2, STAT3, and PTPN11
Jak2-STAT3 signaling pathway	4	1.09*E*−04	LEP, JAK2, STAT3, and PTPN11
IL-6-type cytokine-signal-transduction	3	0.001878	JAK2, STAT3, and PTPN11

**Table 4:** mRNA expression levels related to the JAK2-STAT3 pathway in the mice liver after drug administration ($\bar{x}\pm s$).

Group	Amount (*n*)	LepRb	SOCS-3	JAK2	STAT3
Normal group	5	1.00	1.00	1.00	1.00
Model group	5	0.29 ± 0.05^a^	8.35 ± 2.02^a^	0.34 ± 0.20^a^	0.65 ± 0.11^a^
Positive control group	5	0.65 ± 0.15^b^	7.43 ± 2.21^a^	0.59 ± 0.05^a^	0.99 ± 0.09^b^
High-dose group	5	1.11 ± 0.29^b^	1.90 ± 1.09^bc^	0.93 ± 0.22^b^	1.08 ± 0.19^b^
Medium-dose group	5	0.94 ± 0.23^b^	1.08 ± 0.21^bc^	0.94 ± 0.12^bc^	0.92 ± 0.05^b^
Low-dose group	5	0.31 ± 0.09^acde^	1.26 ± 0.57^bc^	0.88 ± 0.10^bc^	0.71 ± 0.18^ace^
*F*		22.223	33.401	17.036	13.891
*P*		0.001	0.004	0.001	0.001

**Table 5:** mRNA expression levels related to the JAK2-STAT3 pathway in the fat of mice after administration ($\bar{x}\pm s$).

Group	Amount (*n*)	LepRb	SOCS-3	JAK2	STAT3
Normal group	5	1.00	1.00	1.00	1.00
Model group	5	0.05 ± 0.04^a^	2.32 ± 0.17^a^	0.17 ± 0.03^a^	0.29 ± 0.09^a^
Positive control group	5	0.46 ± 0.12^ab^	2.05 ± 0.31^a^	0.28 ± 0.06^a^	0.59 ± 0.11^ab^
High-dose group	5	0.53 ± 0.18^ab^	1.72 ± 0.14^ab^	0.83 ± 0.25^b^	0.60 ± 0.13^ab^
Medium-dose group	5	0.69 ± 0.16^b^	1.17 ± 0.16^bcd^	0.83 ± 0.14^bc^	0.55 ± 0.08^ab^
Low-dose group	5	0.14 ± 0.05^ace^	1.59 ± 0.14^abe^	0.62 ± 0.15^abc^	0.38 ± 0.04^ae^
F		49.125	40.291	29.735	38.566
*P*		0.002	0.004	0.001	0.001

Note: ^a^*P* < 0.05 vs normal group; ^b^*P* < 0.05 vs model group; ^c^*P* < 0.05 vs positive control group; ^d^*P* < 0.05 vs high-dose group; ^e^*P* < 0.05 vs medium-dose group.

## Data Availability

The data used to support the findings of this study are included within the article and the supplementary materials.

## Supplementary Materials

Supplementary Materials Supplementary Table S1: The information of all components of HJJPD. Supplementary Table S2: Targets of 169 components. Supplementary S3: The biological network of HJJPD acts on the proteins related to the AMPK-ACC pathway, LepRb-IRS-PI3K-PDE3B-cAMP pathway, and LepRb-SHP2-MAPKs (ERK1/2) pathway.
