# Supplementary material for: Study on the Effect and Mechanism of Huaji Jianpi Decoction on Simple Obesity
Source: Evid Based Complement Alternat Med. 2022 Apr 28;2022:5494224. doi: 10.1155/2022/5494224 (PMC9071864; doi:10.1155/2022/5494224)
Supplement: Supplementary Materials — Supplementary Table S1: The information of all components of HJJPD. Supplementary Table S2: Targets of 169 components. Supplementary S3: The biological network of HJJPD acts on the proteins related to the AMPK-ACC pathway, LepRb-IRS-PI3K-PDE3B-cAMP pathway, and LepRb-SHP2-MAPKs (ERK1/2) pathway. [file 5494224.f1.zip › 5494224.f1/Supplementary Table 1.docx]

| **Herb name** | **Components ID** | **CAS** | **Components name** | **OB** | **Drug-likeness** |
| --- | --- | --- | --- | --- | --- |
| *pinelliae rhizoma* | MOL005030 | 5561-99-9 | gondoic acid | 30.702942552 | 0.19743 |
| *pinelliae rhizoma* | MOL006936 | 30223-50-8 | 10,13-eicosadienoic | 39.9935540799 | 0.20012 |
| *pinelliae rhizoma* | MOL006967 | 5968-90-1 | beta-D-Ribofuranoside, xanthine-9 | 44.7187854789 | 0.20816 |
| *pinelliae rhizoma* | MOL002714 | 491-67-8 | baicalein | 33.51891869 | 0.20888 |
| *pinelliae rhizoma* | MOL006937 | #N/A | 12,13-epoxy-9-hydroxynonadeca-7,10-dienoic acid | 42.1521761069 | 0.24248 |
| *pinelliae rhizoma* | MOL006957 | #N/A | (3S,6S)-3-(benzyl)-6-(4-hydroxybenzyl)piperazine-2,5-quinone | 46.8888952025 | 0.26989 |
| *pinelliae rhizoma* | MOL000519 | 109664-02-0 | coniferin | 31.1099992042 | 0.32308 |
| *pinelliae rhizoma* | MOL000358 | 83-46-5 | beta-sitosterol | 36.9139058327 | 0.75123 |
| *pinelliae rhizoma* | MOL002776 | 31564-28-0 | Baicalin | 40.1236099599 | 0.75264 |
| *pinelliae rhizoma* | MOL000449 | 83-48-7 | Stigmasterol | 43.8298515785 | 0.75665 |
| *pinelliae rhizoma* | MOL001755 | 67392-96-5 | 24-Ethylcholest-4-en-3-one | 36.0836116436 | 0.75703 |
| *pinelliae rhizoma* | MOL003578 | 469-38-5 | Cycloartenol | 38.685659058 | 0.78093 |
| *pinelliae rhizoma* | MOL002670 | 32728-75-9 | Cavidine | 35.6418304575 | 0.80513 |
| *roasted atractylodis macrocephalae rhizoma* | MOL000072 | 113269-35-5 | 8β-ethoxy atractylenolide Ⅲ | 35.950919284 | 0.21079 |
| *roasted atractylodis macrocephalae rhizoma* | MOL000049 | 61206-10-8 | 3β-acetoxyatractylone | 54.066717067 | 0.21906 |
| *roasted atractylodis macrocephalae rhizoma* | MOL000020 | 113269-39-9 | 12-senecioyl-2E,8E,10E-atractylentriol | 62.39646702 | 0.22294 |
| *roasted atractylodis macrocephalae rhizoma* | MOL000022 | 113269-36-6 | 14-acetyl-12-senecioyl-2E,8Z,10E-atractylentriol | 63.3709182303 | 0.29956 |
| *roasted atractylodis macrocephalae rhizoma* | MOL000021 | 113269-37-7 | 14-acetyl-12-senecioyl-2E,8E,10E-atractylentriol | 60.312870701 | 0.30534 |
| *roasted atractylodis macrocephalae rhizoma* | MOL000028 | 638-95-9 | α-Amyrin | 39.5120897831 | 0.7629 |
| *roasted atractylodis macrocephalae rhizoma* | MOL000033 | 64997-52-0 | (3S,8S,9S,10R,13R,14S,17R)-10,13-dimethyl-17-[(2R,5S)-5-propan-2-yloctan-2-yl]-2,3,4,7,8,9,11,12,14,15,16,17-dodecahydro-1H-cyclopenta[a]phenanthren-3-ol | 36.2284705584 | 0.78288 |
| *roasted coicis semen* | MOL001494 | 544-35-4 | Mandenol | 41.99620045 | 0.19321 |
| *roasted coicis semen* | MOL008121 | 3443-84-3 | 2-Monoolein | 34.2349738128 | 0.29162 |
| *roasted coicis semen* | MOL002882 | 111-03-5 | [(2R)-2,3-dihydroxypropyl] (Z)-octadec-9-enoate | 34.1310775832 | 0.29824 |
| *roasted coicis semen* | MOL002372 | 7683-64-9 | (6Z,10E,14E,18E)-2,6,10,15,19,23-hexamethyltetracosa-2,6,10,14,18,22-hexaene | 33.5459426365 | 0.42436 |
| *roasted coicis semen* | MOL008118 | 29066-43-1 | Coixenolide | 32.3981341983 | 0.42591 |
| *roasted coicis semen* | MOL000953 | 80356-14-5 | CLR | 37.8738975447 | 0.67677 |
| *roasted coicis semen* | MOL000359 | 83-46-5 | sitosterol | 36.91390583 | 0.7512 |
| *roasted coicis semen* | MOL000449 | 83-48-7 | Stigmasterol | 43.8298515785 | 0.75665 |
| *roasted coicis semen* | MOL001323 | 474-40-8 | Sitosterol alpha1 | 43.281270422 | 0.78354 |
| *roasted aurantii fructus iimmaturus* | MOL004328 | 153-18-4 | naringenin | 59.2938977347 | 0.21128 |
| *roasted aurantii fructus iimmaturus* | MOL001941 | 482-44-0 | Ammidin | 34.5485639384 | 0.22355 |
| *roasted aurantii fructus iimmaturus* | MOL005849 | 14259-47-3 | didymin | 38.551386317 | 0.23908 |
| *roasted aurantii fructus iimmaturus* | MOL002914 | 4049-38-1 | Eriodyctiol (flavanone) | 41.3504271334 | 0.2436 |
| *roasted aurantii fructus iimmaturus* | MOL000006 | 491-70-3 | luteolin | 36.1626293429 | 0.24552 |
| *roasted aurantii fructus iimmaturus* | MOL001798 | 13241-33-3 | neohesperidin_qt | 71.1688584844 | 0.27085 |
| *roasted aurantii fructus iimmaturus* | MOL005100 | 520-26-3 | 5,7-dihydroxy-2-(3-hydroxy-4-methoxyphenyl)chroman-4-one | 47.7364369369 | 0.27226 |
| *roasted aurantii fructus iimmaturus* | MOL013433 | 31575-93-6 | prangenin hydrate | 72.6340064072 | 0.28863 |
| *roasted aurantii fructus iimmaturus* | MOL013430 | 2880-49-1 | Prangenin | 43.5973407516 | 0.29428 |
| *roasted aurantii fructus iimmaturus* | MOL013279 | 5631-70-9 | 5,7,4'-Trimethylapigenin | 39.8327233506 | 0.29636 |
| *roasted aurantii fructus iimmaturus* | MOL013437 | 28587-43-1 | 6-Methoxy aurapten | 31.2377683511 | 0.3008 |
| *roasted aurantii fructus iimmaturus* | MOL013436 | 59176-65-7 | isoponcimarin | 63.2776001702 | 0.31316 |
| *roasted aurantii fructus iimmaturus* | MOL013435 | 55916-48-8 | poncimarin | 63.6209280574 | 0.34942 |
| *roasted aurantii fructus iimmaturus* | MOL007879 | 855-97-0 | Tetramethoxyluteolin | 43.6847646189 | 0.37009 |
| *roasted aurantii fructus iimmaturus* | MOL009053 | 4263-87-0 | 4-[(2S,3R)-5-[(E)-3-hydroxyprop-1-enyl]-7-methoxy-3-methylol-2,3-dihydrobenzofuran-2-yl]-2-methoxy-phenol | 50.7551364887 | 0.3948 |
| *roasted aurantii fructus iimmaturus* | MOL013277 | #N/A | Isosinensetin | 51.1516915388 | 0.44149 |
| *roasted aurantii fructus iimmaturus* | MOL001803 | 2306-27-6 | Sinensetin | 50.55684919 | 0.44634 |
| *roasted aurantii fructus iimmaturus* | MOL005828 | 478-01-3 | nobiletin | 61.6694393243 | 0.51652 |
| *roasted aurantii fructus iimmaturus* | MOL013440 | 105279-10-5 | citrusin B | 40.7971664127 | 0.71331 |
| *roasted aurantii fructus iimmaturus* | MOL013428 | 14259-47-3 | isosakuranetin-7-rutinoside | 41.2401275665 | 0.71616 |
| *roasted aurantii fructus iimmaturus* | MOL013276 | 14941-08-3 | poncirin | 36.5460126322 | 0.74202 |
| *roasted aurantii fructus iimmaturus* | MOL013352 | 751-03-1 | Obacunone | 43.2862536493 | 0.76724 |
| *citri reticulatae pericarpium* | MOL004328 | 153-18-4 | naringenin | 59.2938977347 | 0.21128 |
| *citri reticulatae pericarpium* | MOL005100 | 520-26-3 | 5,7-dihydroxy-2-(3-hydroxy-4-methoxyphenyl)chroman-4-one | 47.7364369369 | 0.27226 |
| *citri reticulatae pericarpium* | MOL005815 | #N/A | Citromitin | 86.9040467185 | 0.51439 |
| *citri reticulatae pericarpium* | MOL005828 | 478-01-3 | nobiletin | 61.6694393243 | 0.51652 |
| *citri reticulatae pericarpium* | MOL000359 | 83-46-5 | sitosterol | 36.91390583 | 0.7512 |
| *poria* | MOL000282 | 1105-11-9 | ergosta-7,22E-dien-3beta-ol | 43.507086374 | 0.71939 |
| *poria* | MOL000291 | 137551-39-4 | Poricoic acid B | 30.5246012947 | 0.7463 |
| *poria* | MOL000292 | 151200-89-4 | poricoic acid C | 38.1513578853 | 0.74643 |
| *poria* | MOL000296 | 465-99-6 | hederagenin | 36.9139058327 | 0.75072 |
| *poria* | MOL000290 | 137551-38-3 | Poricoic acid A | 30.60694619 | 0.76152 |
| *poria* | MOL000279 | 516-37-0 | Cerevisterol | 37.96382825 | 0.77061 |
| *poria* | MOL000275 | 24160-36-9 | trametenolic acid | 38.7115000211 | 0.80199 |
| *poria* | MOL000287 | #N/A | 3beta-Hydroxy-24-methylene-8-lanostene-21-oic acid | 38.6999140072 | 0.8095 |
| *poria* | MOL000289 | 29070-92-6 | pachymic acid | 33.6279195687 | 0.81076 |
| *poria* | MOL000276 | 77012-31-8 | 7,9(11)-dehydropachymic acid | 35.1058909952 | 0.81091 |
| *poria* | MOL000283 | #N/A | Ergosterol peroxide | 40.3626804838 | 0.81255 |
| *poria* | MOL000273 | #N/A | (2R)-2-[(3S,5R,10S,13R,14R,16R,17R)-3,16-dihydroxy-4,4,10,13,14-pentamethyl-2,3,5,6,12,15,16,17-octahydro-1H-cyclopenta[a]phenanthren-17-yl]-6-methylhept-5-enoic acid | 30.93214234 | 0.81281 |
| *poria* | MOL000280 | 6754-16-1 | (2R)-2-[(3S,5R,10S,13R,14R,16R,17R)-3,16-dihydroxy-4,4,10,13,14-pentamethyl-2,3,5,6,12,15,16,17-octahydro-1H-cyclopenta[a]phenanthren-17-yl]-5-isopropyl-hex-5-enoic acid | 31.0720566481 | 0.81528 |
| *poria* | MOL000285 | 465-18-9 | (2R)-2-[(5R,10S,13R,14R,16R,17R)-16-hydroxy-3-keto-4,4,10,13,14-pentamethyl-1,2,5,6,12,15,16,17-octahydrocyclopenta[a]phenanthren-17-yl]-5-isopropyl-hex-5-enoic acid | 38.2551579991 | 0.82014 |
| *poria* | MOL000300 | #N/A | dehydroeburicoic acid | 44.1722986724 | 0.83458 |
| *nelumbinis folium* | MOL006405 | 66277-20-1 | (1S)-1-(4-hydroxybenzyl)-2-methyl-3,4-dihydro-1H-isoquinoline-6,7-diol | 67.1397933197 | 0.23227 |
| *nelumbinis folium* | MOL007207 | 2196-60-3 | Machiline | 79.641604324 | 0.23513 |
| *nelumbinis folium* | MOL000422 | 520-18-3 | kaempferol | 41.8822495352 | 0.24066 |
| *nelumbinis folium* | MOL000073 | 35323-91-2 | ent-Epicatechin | 48.95984114 | 0.24162 |
| *nelumbinis folium* | MOL000096 | 154-23-4 | (-)-catechin | 49.6763868 | 0.24162 |
| *nelumbinis folium* | MOL007214 | 69256-15-1 | (+)-Leucocyanidin | 37.6062475031 | 0.27124 |
| *nelumbinis folium* | MOL000098 | 73123-10-1 | quercetin | 46.4333481195 | 0.27525 |
| *nelumbinis folium* | MOL007206 | 524-20-9 | Armepavine | 69.3090585966 | 0.28773 |
| *nelumbinis folium* | MOL000354 | 480-19-3 | isorhamnetin | 49.604377053 | 0.306 |
| *nelumbinis folium* | MOL007217 | 491-52-1 | leucodelphinidin | 30.0241001456 | 0.30628 |
| *nelumbinis folium* | MOL007210 | 3153-55-7 | o-Nornuciferine | 33.5158316274 | 0.36421 |
| *nelumbinis folium* | MOL007213 | 475-83-2 | Nuciferin | 34.4310288295 | 0.40475 |
| *nelumbinis folium* | MOL007218 | 548-08-3 | Remerin | 40.7549157795 | 0.5208 |
| *nelumbinis folium* | MOL000359 | 83-46-5 | sitosterol | 36.91390583 | 0.7512 |
| *nelumbinis folium* | MOL003578 | 469-38-5 | Cycloartenol | 38.685659058 | 0.78093 |
| *astragali radix* | MOL000392 | 485-72-3 | formononetin | 69.6738806088 | 0.21202 |
| *astragali radix* | MOL000422 | 520-18-3 | kaempferol | 41.8822495352 | 0.24066 |
| *astragali radix* | MOL000417 | 20575-57-9 | Calycosin | 47.7518278266 | 0.24278 |
| *astragali radix* | MOL000438 | 64474-51-7 | (3R)-3-(2-hydroxy-3,4-dimethoxyphenyl)chroman-7-ol | 67.6674794931 | 0.26479 |
| *astragali radix* | MOL000098 | 73123-10-1 | quercetin | 46.4333481195 | 0.27525 |
| *astragali radix* | MOL000239 | 3301-49-3 | Jaranol | 50.8288167701 | 0.29148 |
| *astragali radix* | MOL000378 | #N/A | 7-O-methylisomucronulatol | 74.6861375238 | 0.29792 |
| *astragali radix* | MOL000354 | 480-19-3 | isorhamnetin | 49.604377053 | 0.306 |
| *astragali radix* | MOL000380 | 73340-41-7 | (6aR,11aR)-9,10-dimethoxy-6a,11a-dihydro-6H-benzofurano[3,2-c]chromen-3-ol | 64.2554545232 | 0.42486 |
| *astragali radix* | MOL000371 | #N/A | 3,9-di-O-methylnissolin | 53.7415267252 | 0.47573 |
| *astragali radix* | MOL000442 | #N/A | 1,7-Dihydroxy-3,9-dimethoxy pterocarpene | 39.0454111203 | 0.47943 |
| *astragali radix* | MOL000439 | #N/A | isomucronulatol-7,2'-di-O-glucosiole | 49.281055391 | 0.62065 |
| *astragali radix* | MOL000387 | 73536-69-3 | Bifendate | 31.0978239059 | 0.66553 |
| *astragali radix* | MOL000374 | #N/A | 5'-hydroxyiso-muronulatol-2',5'-di-O-glucoside | 41.7176657373 | 0.69251 |
| *astragali radix* | MOL000433 | 33609-88-0 | FA | 68.9604362164 | 0.7057 |
| *astragali radix* | MOL000296 | 465-99-6 | hederagenin | 36.9139058327 | 0.75072 |
| *astragali radix* | MOL000211 | 472-15-1 | Mairin | 55.3770733787 | 0.7761 |
| *astragali radix* | MOL000033 | 64997-52-0 | (3S,8S,9S,10R,13R,14S,17R)-10,13-dimethyl-17-[(2R,5S)-5-propan-2-yloctan-2-yl]-2,3,4,7,8,9,11,12,14,15,16,17-dodecahydro-1H-cyclopenta[a]phenanthren-3-ol | 36.2284705584 | 0.78288 |
| *astragali radix* | MOL000379 | 94367-42-7 | 9,10-dimethoxypterocarpan-3-O-β-D-glucoside | 36.736688011 | 0.9243 |
| *alismatis rhizoma* | MOL002464 | 107380-08-5 | 1-Monolinolein | 37.1766283612 | 0.30249 |
| *alismatis rhizoma* | MOL000359 | 83-46-5 | sitosterol | 36.91390583 | 0.7512 |
| *alismatis rhizoma* | MOL000849 | 115333-90-9 | 16β-methoxyalisol B monoacetate | 32.4272410552 | 0.7679 |
| *alismatis rhizoma* | MOL000831 | 26575-95-1 | Alisol B monoacetate | 35.5762362126 | 0.80629 |
| *alismatis rhizoma* | MOL000862 | 19865-76-0 | [(1S,3R)-1-[(2R)-3,3-dimethyloxiran-2-yl]-3-[(5R,8S,9S,10S,11S,14R)-11-hydroxy-4,4,8,10,14-pentamethyl-3-oxo-1,2,5,6,7,9,11,12,15,16-decahydrocyclopenta[a]phenanthren-17-yl]butyl] acetate | 35.5762362126 | 0.80765 |
| *alismatis rhizoma* | MOL000854 | 30489-27-1 | alisol C | 32.7001692128 | 0.81507 |
| *alismatis rhizoma* | MOL000830 | #N/A | Alisol B | 34.4730730771 | 0.81706 |
| *alismatis rhizoma* | MOL000832 | 26575-95-1 | alisol,b,23-acetate | 32.516216012 | 0.81841 |
| *alismatis rhizoma* | MOL000853 | 18649-93-9 | alisol B | 36.7603806659 | 0.81993 |
| *alismatis rhizoma* | MOL000856 | 26575-93-9 | alisol C monoacetate | 33.0635894743 | 0.82763 |
