# Supplementary material for: Study on the Effect and Mechanism of Huaji Jianpi Decoction on Simple Obesity
Source: Evid Based Complement Alternat Med. 2022 Apr 28;2022:5494224. doi: 10.1155/2022/5494224 (PMC9071864; doi:10.1155/2022/5494224)
Supplement: Supplementary Materials — Supplementary Table S1: The information of all components of HJJPD. Supplementary Table S2: Targets of 169 components. Supplementary S3: The biological network of HJJPD acts on the proteins related to the AMPK-ACC pathway, LepRb-IRS-PI3K-PDE3B-cAMP pathway, and LepRb-SHP2-MAPKs (ERK1/2) pathway. [file 5494224.f1.zip › 5494224.f1/Supplementary Table 2.docx]

| **Components ID** | **Targets** | **Symbol** |
| --- | --- | --- |
| MOL000006 | Transcription factor AP-1 | AP1 |
| MOL000006 | Amyloid beta A4 protein | APP |
| MOL000006 | Androgen receptor | AR |
| MOL000006 | Carbonic anhydrase II | CA2 |
| MOL000006 | Caspase-7 | CASP7 |
| MOL000006 | Cyclin-A2 | CCNA2 |
| MOL000006 | Dipeptidyl peptidase IV | CD26 |
| MOL000006 | Cell division protein kinase 2 | Cdk2 |
| MOL000006 | Cell division protein kinase 4 | Cdk4 |
| MOL000006 | Prostaglandin G/H synthase 1 | COX1 |
| MOL000006 | Epidermal growth factor receptor | EGFR |
| MOL000006 | Mitogen-activated protein kinase 1 | ERK2 |
| MOL000006 | Estrogen receptor | ESR1 |
| MOL000006 | Estrogen receptor beta | ESR2 |
| MOL000006 | Glycogen synthase kinase-3 beta | GSK3B |
| MOL000006 | Glutathione S-transferase P | GSTP1 |
| MOL000006 | Heme oxygenase 1 | HMOX1 |
| MOL000006 | Heat shock protein HSP 90 | HSP90 |
| MOL000006 | Interferon gamma | IFNG |
| MOL000006 | Interleukin-2 | IL2 |
| MOL000006 | Interleukin-6 | IL6 |
| MOL000006 | Nitric oxide synthase, inducible | iNOS |
| MOL000006 | Insulin receptor | INSR |
| MOL000006 | Mitogen-activated protein kinase 14 | MAPK14 |
| MOL000006 | Hepatocyte growth factor receptor | MET |
| MOL000006 | Interstitial collagenase | MMP1 |
| MOL000006 | Nuclear receptor coactivator 2 | NCOA2 |
| MOL000006 | Cellular tumor antigen p53 | P53_HUMAN |
| MOL000006 | Phosphatidylinositol-4,5-bisphosphate 3-kinase catalytic subunit, gamma isoform | PIK3CG |
| MOL000006 | Proto-oncogene serine/threonine-protein kinase Pim-1 | PIM1 |
| MOL000006 | Peroxisome proliferator activated receptor gamma | PPARG |
| MOL000006 | mRNA of PKA Catalytic Subunit C-alpha | PRKACA |
| MOL000006 | Trypsin-1 | PRSS1 |
| MOL000006 | Prostaglandin G/H synthase 2 | PTGS2 |
| MOL000006 | mRNA of Protein-tyrosine phosphatase, non-receptor type 1 | PTPN1 |
| MOL000006 | Retinoblastoma-associated protein | RB1 |
| MOL000006 | Tumor necrosis factor | TNF |
| MOL000006 | DNA topoisomerase 1 | TOP1 |
| MOL000006 | Vascular endothelial growth factor A | VEGFA |
| MOL000006 | Xanthine dehydrogenase/oxidase | XD |
| MOL000022 | Estrogen receptor | ESR1 |
| MOL000022 | Thrombin | F2 |
| MOL000022 | Peroxisome proliferator activated receptor gamma | PPARG |
| MOL000022 | Prostaglandin G/H synthase 2 | PTGS2 |
| MOL000033 | Androgen receptor | AR |
| MOL000033 | Estrogen receptor | ESR1 |
| MOL000033 | Progesterone receptor | PGR |
| MOL000049 | Acetylcholinesterase | ACHE |
| MOL000049 | Alpha-1A adrenergic receptor | ADRA1A |
| MOL000049 | Beta-2 adrenergic receptor | ADRB2 |
| MOL000049 | Androgen receptor | AR |
| MOL000049 | Dipeptidyl peptidase IV | CD26 |
| MOL000049 | Cell division protein kinase 2 | Cdk2 |
| MOL000049 | Muscarinic acetylcholine receptor M1 | CHRM1 |
| MOL000049 | Muscarinic acetylcholine receptor M2 | CHRM2 |
| MOL000049 | Muscarinic acetylcholine receptor M3 | Chrm3 |
| MOL000049 | Neuronal acetylcholine receptor protein, alpha-7 chain | CHRNA7 |
| MOL000049 | Estrogen receptor | ESR1 |
| MOL000049 | Estrogen receptor beta | ESR2 |
| MOL000049 | Thrombin | F2 |
| MOL000049 | Gamma-aminobutyric acid receptor subunit alpha-1 | GABRA1 |
| MOL000049 | Nitric oxide synthase, inducible | iNOS |
| MOL000049 | Mu-type opioid receptor | MOR-1 |
| MOL000049 | Nitric-oxide synthase, endothelial | NOS |
| MOL000049 | Prostaglandin G/H synthase 2 | PTGS2 |
| MOL000049 | Retinoic acid receptor RXR-alpha | RXRA |
| MOL000049 | Sodium channel protein type 5 subunit alpha | SCN5A |
| MOL000072 | Acetylcholinesterase | ACHE |
| MOL000072 | Androgen receptor | AR |
| MOL000072 | Dipeptidyl peptidase IV | CD26 |
| MOL000072 | Neuronal acetylcholine receptor protein, alpha-7 chain | CHRNA7 |
| MOL000072 | Estrogen receptor | ESR1 |
| MOL000072 | Thrombin | F2 |
| MOL000072 | Gamma-aminobutyric acid receptor subunit alpha-1 | GABRA1 |
| MOL000072 | Nitric oxide synthase, inducible | iNOS |
| MOL000072 | Nuclear receptor coactivator 1 | NCOA1 |
| MOL000072 | Nuclear receptor coactivator 2 | NCOA2 |
| MOL000072 | Nitric-oxide synthase, endothelial | NOS |
| MOL000072 | Prostaglandin G/H synthase 2 | PTGS2 |
| MOL000073 | Androgen receptor | AR |
| MOL000073 | Carbonic anhydrase II | CA2 |
| MOL000073 | Cyclin-A2 | CCNA2 |
| MOL000073 | Dipeptidyl peptidase IV | CD26 |
| MOL000073 | Cell division protein kinase 2 | Cdk2 |
| MOL000073 | Prostaglandin G/H synthase 1 | COX1 |
| MOL000073 | Estrogen receptor | ESR1 |
| MOL000073 | Estrogen receptor beta | ESR2 |
| MOL000073 | Glycogen synthase kinase-3 beta | GSK3B |
| MOL000073 | Heat shock protein HSP 90 | HSP90 |
| MOL000073 | Nitric oxide synthase, inducible | iNOS |
| MOL000073 | Beta-lactamase | LACTB |
| MOL000073 | Mitogen-activated protein kinase 14 | MAPK14 |
| MOL000073 | Proto-oncogene serine/threonine-protein kinase Pim-1 | PIM1 |
| MOL000073 | Peroxisome proliferator activated receptor gamma | PPARG |
| MOL000073 | mRNA of PKA Catalytic Subunit C-alpha | PRKACA |
| MOL000073 | Prostaglandin G/H synthase 2 | PTGS2 |
| MOL000073 | mRNA of Protein-tyrosine phosphatase, non-receptor type 1 | PTPN1 |
| MOL000096 | Androgen receptor | AR |
| MOL000096 | Carbonic anhydrase II | CA2 |
| MOL000096 | Calmodulin | CALM1 |
| MOL000096 | Cyclin-A2 | CCNA2 |
| MOL000096 | Dipeptidyl peptidase IV | CD26 |
| MOL000096 | Cell division protein kinase 2 | Cdk2 |
| MOL000096 | Prostaglandin G/H synthase 1 | COX1 |
| MOL000096 | Estrogen receptor | ESR1 |
| MOL000096 | Estrogen receptor beta | ESR2 |
| MOL000096 | Fatty acid synthase | FAS |
| MOL000096 | Glycogen synthase kinase-3 beta | GSK3B |
| MOL000096 | Heat shock protein HSP 90 | HSP90 |
| MOL000096 | Nitric oxide synthase, inducible | iNOS |
| MOL000096 | Beta-lactamase | LACTB |
| MOL000096 | Mitogen-activated protein kinase 14 | MAPK14 |
| MOL000096 | Nuclear receptor coactivator 2 | NCOA2 |
| MOL000096 | Proto-oncogene serine/threonine-protein kinase Pim-1 | PIM1 |
| MOL000096 | Peroxisome proliferator activated receptor gamma | PPARG |
| MOL000096 | mRNA of PKA Catalytic Subunit C-alpha | PRKACA |
| MOL000096 | Trypsin-1 | PRSS1 |
| MOL000096 | Prostaglandin G/H synthase 2 | PTGS2 |
| MOL000096 | mRNA of Protein-tyrosine phosphatase, non-receptor type 1 | PTPN1 |
| MOL000098 | Acetyl-CoA carboxylase 1 | ACC1 |
| MOL000098 | Acetylcholinesterase | ACHE |
| MOL000098 | Beta-2 adrenergic receptor | ADRB2 |
| MOL000098 | Aryl hydrocarbon receptor | AHR |
| MOL000098 | Aldose reductase | ALDR1 |
| MOL000098 | Arachidonate 5-lipoxygenase | ALOX5 |
| MOL000098 | Transcription factor AP-1 | AP1 |
| MOL000098 | Androgen receptor | AR |
| MOL000098 | Apoptosis regulator Bcl-2 | BCL2 |
| MOL000098 | 78 kDa glucose-regulated protein | BiP |
| MOL000098 | Carbonic anhydrase II | CA2 |
| MOL000098 | C-C motif chemokine 2 | CCL2 |
| MOL000098 | Cyclin-A2 | CCNA2 |
| MOL000098 | Dipeptidyl peptidase IV | CD26 |
| MOL000098 | Cell division protein kinase 2 | Cdk2 |
| MOL000098 | Serine/threonine-protein kinase Chk1 | Chk1 |
| MOL000098 | Collagen alpha-1(I) chain | COL1A1 |
| MOL000098 | Collagen alpha-1(III) chain | COL3A1 |
| MOL000098 | Prostaglandin G/H synthase 1 | COX1 |
| MOL000098 | Cell division control protein 2 homolog | CRK2 |
| MOL000098 | Cathepsin D | CTSD |
| MOL000098 | Cytochrome P450 1A2 | CYP1A2 |
| MOL000098 | Prostatic acid phosphatase | D3Y5P |
| MOL000098 | Pro-epidermal growth factor | EGF |
| MOL000098 | Epidermal growth factor receptor | EGFR |
| MOL000098 | Mitogen-activated protein kinase 1 | ERK2 |
| MOL000098 | Estrogen receptor | ESR1 |
| MOL000098 | Estrogen receptor beta | ESR2 |
| MOL000098 | Coagulation factor Xa | F10 |
| MOL000098 | Thrombin | F2 |
| MOL000098 | Coagulation factor VII | F7 |
| MOL000098 | Gamma-aminobutyric acid receptor subunit alpha-1 | GABRA1 |
| MOL000098 | Gap junction alpha-1 protein | GJA1 |
| MOL000098 | Glycogen synthase kinase-3 beta | GSK3B |
| MOL000098 | Glutathione S-transferase Mu 1 | GSTM1 |
| MOL000098 | Glutathione S-transferase Mu 2 | GSTM2 |
| MOL000098 | Glutathione S-transferase P | GSTP1 |
| MOL000098 | DNA gyrase subunit B | GYRB |
| MOL000098 | Heme oxygenase 1 | HMOX1 |
| MOL000098 | Heat shock protein HSP 90 | HSP90 |
| MOL000098 | Interferon gamma | IFNG |
| MOL000098 | Interleukin-1 beta | IL1B |
| MOL000098 | Interleukin-2 | IL2 |
| MOL000098 | Interleukin-6 | IL6 |
| MOL000098 | Nitric oxide synthase, inducible | iNOS |
| MOL000098 | Insulin receptor | INSR |
| MOL000098 | Potassium voltage-gated channel subfamily H member 2 | KCNH2 |
| MOL000098 | Beta-lactamase | LACTB |
| MOL000098 | Amine oxidase [flavin-containing] B | MAOB |
| MOL000098 | Mitogen-activated protein kinase 14 | MAPK14 |
| MOL000098 | Maltase-glucoamylase, intestinal | MGAM |
| MOL000098 | Interstitial collagenase | MMP1 |
| MOL000098 | Stromelysin-1 | MMP3 |
| MOL000098 | Myeloperoxidase | MPO |
| MOL000098 | Nuclear receptor coactivator 2 | NCOA2 |
| MOL000098 | Cytochrome P450 3A4 | NF-25 |
| MOL000098 | Nitric-oxide synthase, endothelial | NOS |
| MOL000098 | NAD(P)H dehydrogenase [quinone] 1 | NQO1 |
| MOL000098 | Ornithine decarboxylase | ODC |
| MOL000098 | Cellular tumor antigen p53 | P53_HUMAN |
| MOL000098 | Phosphatidylinositol-4,5-bisphosphate 3-kinase catalytic subunit, gamma isoform | PIK3CG |
| MOL000098 | Proto-oncogene serine/threonine-protein kinase Pim-1 | PIM1 |
| MOL000098 | Tissue-type plasminogen activator | PLAT |
| MOL000098 | Urokinase-type plasminogen activator | PLAU |
| MOL000098 | Serum paraoxonase/arylesterase 1 | PON1 |
| MOL000098 | NADPH--cytochrome P450 reductase | POR |
| MOL000098 | Peroxisome proliferator activated receptor gamma | PPARG |
| MOL000098 | mRNA of PKA Catalytic Subunit C-alpha | PRKACA |
| MOL000098 | Trypsin-1 | PRSS1 |
| MOL000098 | Prostaglandin E2 receptor EP3 subtype | PTGER3 |
| MOL000098 | Prostaglandin G/H synthase 2 | PTGS2 |
| MOL000098 | mRNA of Protein-tyrosine phosphatase, non-receptor type 1 | PTPN1 |
| MOL000098 | Retinoblastoma-associated protein | RB1 |
| MOL000098 | Retinoic acid receptor RXR-alpha | RXRA |
| MOL000098 | Sodium channel protein type 5 subunit alpha | SCN5A |
| MOL000098 | E-selectin | SELE |
| MOL000098 | Superoxide dismutase [Cu-Zn] | SOD |
| MOL000098 | Estrogen sulfotransferase | SULT1E1 |
| MOL000098 | Tissue factor | TF |
| MOL000098 | Thrombomodulin | THBD |
| MOL000098 | Tumor necrosis factor | TNF |
| MOL000098 | DNA topoisomerase 1 | TOP1 |
| MOL000098 | DNA topoisomerase II | TOP2 |
| MOL000098 | Vascular cell adhesion protein 1 | VCAM1 |
| MOL000098 | Vascular endothelial growth factor A | VEGFA |
| MOL000098 | Xanthine dehydrogenase/oxidase | XD |
| MOL000211 | Androgen receptor | AR |
| MOL000211 | Estrogen receptor | ESR1 |
| MOL000211 | Glucocorticoid receptor | NR3C1 |
| MOL000211 | Progesterone receptor | PGR |
| MOL000239 | Androgen receptor | AR |
| MOL000239 | Carbonic anhydrase II | CA2 |
| MOL000239 | Calmodulin | CALM1 |
| MOL000239 | Cyclin-A2 | CCNA2 |
| MOL000239 | Dipeptidyl peptidase IV | CD26 |
| MOL000239 | Cell division protein kinase 2 | Cdk2 |
| MOL000239 | Serine/threonine-protein kinase Chk1 | Chk1 |
| MOL000239 | Prostaglandin G/H synthase 1 | COX1 |
| MOL000239 | Estrogen receptor | ESR1 |
| MOL000239 | Estrogen receptor beta | ESR2 |
| MOL000239 | Glycogen synthase kinase-3 beta | GSK3B |
| MOL000239 | Heat shock protein HSP 90 | HSP90 |
| MOL000239 | Nitric oxide synthase, inducible | iNOS |
| MOL000239 | Mitogen-activated protein kinase 14 | MAPK14 |
| MOL000239 | Nuclear receptor coactivator 2 | NCOA2 |
| MOL000239 | Proto-oncogene serine/threonine-protein kinase Pim-1 | PIM1 |
| MOL000239 | Peroxisome proliferator activated receptor gamma | PPARG |
| MOL000239 | Trypsin-1 | PRSS1 |
| MOL000239 | Prostaglandin G/H synthase 2 | PTGS2 |
| MOL000239 | mRNA of Protein-tyrosine phosphatase, non-receptor type 1 | PTPN1 |
| MOL000239 | Sodium channel protein type 5 subunit alpha | SCN5A |
| MOL000273 | Androgen receptor | AR |
| MOL000273 | Nuclear receptor coactivator 2 | NCOA2 |
| MOL000273 | Glucocorticoid receptor | NR3C1 |
| MOL000273 | Mineralocorticoid receptor | NR3C2 |
| MOL000275 | Androgen receptor | AR |
| MOL000275 | Estrogen receptor | ESR1 |
| MOL000275 | Glucocorticoid receptor | NR3C1 |
| MOL000275 | Mineralocorticoid receptor | NR3C2 |
| MOL000276 | Glucocorticoid receptor | NR3C1 |
| MOL000279 | Androgen receptor | AR |
| MOL000279 | Estrogen receptor | ESR1 |
| MOL000279 | Glucocorticoid receptor | NR3C1 |
| MOL000279 | Mineralocorticoid receptor | NR3C2 |
| MOL000280 | Androgen receptor | AR |
| MOL000280 | Glucocorticoid receptor | NR3C1 |
| MOL000282 | Androgen receptor | AR |
| MOL000282 | Estrogen receptor | ESR1 |
| MOL000282 | Glucocorticoid receptor | NR3C1 |
| MOL000282 | Progesterone receptor | PGR |
| MOL000283 | Androgen receptor | AR |
| MOL000283 | Estrogen receptor | ESR1 |
| MOL000283 | Glucocorticoid receptor | NR3C1 |
| MOL000283 | Progesterone receptor | PGR |
| MOL000287 | Androgen receptor | AR |
| MOL000287 | Glucocorticoid receptor | NR3C1 |
| MOL000289 | Glucocorticoid receptor | NR3C1 |
| MOL000290 | Androgen receptor | AR |
| MOL000290 | Glucocorticoid receptor | NR3C1 |
| MOL000291 | Androgen receptor | AR |
| MOL000291 | Glucocorticoid receptor | NR3C1 |
| MOL000292 | Androgen receptor | AR |
| MOL000296 | Acetylcholinesterase | ACHE |
| MOL000296 | Alcohol dehydrogenase 1B | ADH1B |
| MOL000296 | Alcohol dehydrogenase 1C | ADH1C |
| MOL000296 | Alpha-1B adrenergic receptor | ADRA1B |
| MOL000296 | Androgen receptor | AR |
| MOL000296 | Carbonic anhydrase II | CA2 |
| MOL000296 | Dipeptidyl peptidase IV | CD26 |
| MOL000296 | Muscarinic acetylcholine receptor M1 | CHRM1 |
| MOL000296 | Muscarinic acetylcholine receptor M2 | CHRM2 |
| MOL000296 | Muscarinic acetylcholine receptor M3 | Chrm3 |
| MOL000296 | Nicotinate-nucleotide--dimethylbenzimidazole phosphoribosyltransferase | cobT |
| MOL000296 | Prostaglandin G/H synthase 1 | COX1 |
| MOL000296 | Cytochrome P450-cam | CYP101A1 |
| MOL000296 | Estrogen receptor | ESR1 |
| MOL000296 | Thrombin | F2 |
| MOL000296 | Gamma-aminobutyric acid receptor subunit alpha-1 | GABRA1 |
| MOL000296 | Gamma-aminobutyric-acid receptor alpha-2 subunit | GABRA2 |
| MOL000296 | Gamma-aminobutyric-acid receptor alpha-3 subunit | GABRA3 |
| MOL000296 | Gamma-aminobutyric-acid receptor alpha-5 subunit | GABRA5 |
| MOL000296 | Gamma-aminobutyric-acid receptor subunit alpha-6 | GABRA6 |
| MOL000296 | Glutamate receptor 2 | GRIA2 |
| MOL000296 | Ig gamma-1 chain C region | IGHG1 |
| MOL000296 | Lysozyme | LYZ |
| MOL000296 | Sodium-dependent noradrenaline transporter | NAT1 |
| MOL000296 | Nuclear receptor coactivator 2 | NCOA2 |
| MOL000296 | Nitric-oxide synthase, endothelial | NOS |
| MOL000296 | Glucocorticoid receptor | NR3C1 |
| MOL000296 | CGMP-inhibited 3',5'-cyclic phosphodiesterase A | PDE3A |
| MOL000296 | Progesterone receptor | PGR |
| MOL000296 | Peroxisome proliferator activated receptor gamma | PPARG |
| MOL000296 | Trypsin-1 | PRSS1 |
| MOL000296 | Prostaglandin G/H synthase 2 | PTGS2 |
| MOL000296 | Retinoic acid receptor RXR-alpha | RXRA |
| MOL000296 | Sodium channel protein type 5 subunit alpha | SCN5A |
| MOL000354 | Acetylcholinesterase | ACHE |
| MOL000354 | Aldose reductase | ALDR1 |
| MOL000354 | Androgen receptor | AR |
| MOL000354 | Carbonic anhydrase II | CA2 |
| MOL000354 | Calmodulin | CALM1 |
| MOL000354 | Cyclin-A2 | CCNA2 |
| MOL000354 | Dipeptidyl peptidase IV | CD26 |
| MOL000354 | Cell division protein kinase 2 | Cdk2 |
| MOL000354 | Serine/threonine-protein kinase Chk1 | Chk1 |
| MOL000354 | Prostaglandin G/H synthase 1 | COX1 |
| MOL000354 | Cytochrome P450-cam | CYP101A1 |
| MOL000354 | Estrogen receptor | ESR1 |
| MOL000354 | Estrogen receptor beta | ESR2 |
| MOL000354 | Thrombin | F2 |
| MOL000354 | Coagulation factor VII | F7 |
| MOL000354 | Gamma-aminobutyric acid receptor subunit alpha-1 | GABRA1 |
| MOL000354 | Glutamate receptor 2 | GRIA2 |
| MOL000354 | Glycogen synthase kinase-3 beta | GSK3B |
| MOL000354 | Heat shock protein HSP 90 | HSP90 |
| MOL000354 | Nitric oxide synthase, inducible | iNOS |
| MOL000354 | Beta-lactamase | LACTB |
| MOL000354 | Amine oxidase [flavin-containing] B | MAOB |
| MOL000354 | Mitogen-activated protein kinase 14 | MAPK14 |
| MOL000354 | Nuclear receptor coactivator 1 | NCOA1 |
| MOL000354 | Nuclear receptor coactivator 2 | NCOA2 |
| MOL000354 | Nitric-oxide synthase, endothelial | NOS |
| MOL000354 | Phosphatidylinositol-4,5-bisphosphate 3-kinase catalytic subunit, gamma isoform | PIK3CG |
| MOL000354 | Proto-oncogene serine/threonine-protein kinase Pim-1 | PIM1 |
| MOL000354 | Peroxisome proliferator activated receptor delta | PPARD |
| MOL000354 | Peroxisome proliferator activated receptor gamma | PPARG |
| MOL000354 | mRNA of PKA Catalytic Subunit C-alpha | PRKACA |
| MOL000354 | Trypsin-1 | PRSS1 |
| MOL000354 | Prostaglandin G/H synthase 2 | PTGS2 |
| MOL000354 | mRNA of Protein-tyrosine phosphatase, non-receptor type 1 | PTPN1 |
| MOL000354 | Glycogen phosphorylase, muscle form | PYGM |
| MOL000354 | Xanthine dehydrogenase/oxidase | XD |
| MOL000358 | Acetylcholinesterase | ACHE |
| MOL000358 | Alpha-1A adrenergic receptor | ADRA1A |
| MOL000358 | Alpha-1B adrenergic receptor | ADRA1B |
| MOL000358 | Beta-2 adrenergic receptor | ADRB2 |
| MOL000358 | Transcription factor AP-1 | AP1 |
| MOL000358 | Androgen receptor | AR |
| MOL000358 | Apoptosis regulator Bcl-2 | BCL2 |
| MOL000358 | Carbonic anhydrase II | CA2 |
| MOL000358 | Cyclin-A2 | CCNA2 |
| MOL000358 | Dipeptidyl peptidase IV | CD26 |
| MOL000358 | Cell division protein kinase 2 | Cdk2 |
| MOL000358 | Serine/threonine-protein kinase Chk1 | Chk1 |
| MOL000358 | Muscarinic acetylcholine receptor M1 | CHRM1 |
| MOL000358 | Muscarinic acetylcholine receptor M2 | CHRM2 |
| MOL000358 | Muscarinic acetylcholine receptor M3 | Chrm3 |
| MOL000358 | Muscarinic acetylcholine receptor M4 | CHRM4 |
| MOL000358 | Neuronal acetylcholine receptor subunit alpha-2 | CHRNA2 |
| MOL000358 | Neuronal acetylcholine receptor protein, alpha-7 chain | CHRNA7 |
| MOL000358 | Prostaglandin G/H synthase 1 | COX1 |
| MOL000358 | Cytochrome P450-cam | CYP101A1 |
| MOL000358 | Dopamine D1 receptor | DRD1 |
| MOL000358 | Estrogen receptor | ESR1 |
| MOL000358 | Estrogen receptor beta | ESR2 |
| MOL000358 | Thrombin | F2 |
| MOL000358 | Gamma-aminobutyric acid receptor subunit alpha-1 | GABRA1 |
| MOL000358 | Gamma-aminobutyric-acid receptor alpha-2 subunit | GABRA2 |
| MOL000358 | Gamma-aminobutyric-acid receptor alpha-3 subunit | GABRA3 |
| MOL000358 | Gamma-aminobutyric-acid receptor alpha-5 subunit | GABRA5 |
| MOL000358 | Glycogen synthase kinase-3 beta | GSK3B |
| MOL000358 | Heat shock protein HSP 90 | HSP90 |
| MOL000358 | 5-hydroxytryptamine 2A receptor | HTR2A |
| MOL000358 | Nitric oxide synthase, inducible | iNOS |
| MOL000358 | Potassium voltage-gated channel subfamily H member 2 | KCNH2 |
| MOL000358 | Beta-lactamase | LACTB |
| MOL000358 | Microtubule-associated protein 2 | MAP2 |
| MOL000358 | Mitogen-activated protein kinase 14 | MAPK14 |
| MOL000358 | Mu-type opioid receptor | MOR-1 |
| MOL000358 | Nuclear receptor coactivator 2 | NCOA2 |
| MOL000358 | Glucocorticoid receptor | NR3C1 |
| MOL000358 | CGMP-inhibited 3',5'-cyclic phosphodiesterase A | PDE3A |
| MOL000358 | Progesterone receptor | PGR |
| MOL000358 | Phosphatidylinositol-4,5-bisphosphate 3-kinase catalytic subunit, gamma isoform | PIK3CG |
| MOL000358 | Proto-oncogene serine/threonine-protein kinase Pim-1 | PIM1 |
| MOL000358 | Serum paraoxonase/arylesterase 1 | PON1 |
| MOL000358 | Peroxisome proliferator activated receptor gamma | PPARG |
| MOL000358 | mRNA of PKA Catalytic Subunit C-alpha | PRKACA |
| MOL000358 | Trypsin-1 | PRSS1 |
| MOL000358 | Prostaglandin G/H synthase 2 | PTGS2 |
| MOL000358 | mRNA of Protein-tyrosine phosphatase, non-receptor type 1 | PTPN1 |
| MOL000358 | Sodium channel protein type 5 subunit alpha | SCN5A |
| MOL000358 | Sodium-dependent serotonin transporter | SLC6A4 |
| MOL000359 | Androgen receptor | AR |
| MOL000359 | Estrogen receptor | ESR1 |
| MOL000359 | Nuclear receptor coactivator 2 | NCOA2 |
| MOL000359 | Glucocorticoid receptor | NR3C1 |
| MOL000359 | Mineralocorticoid receptor | NR3C2 |
| MOL000359 | Progesterone receptor | PGR |
| MOL000371 | Acetylcholinesterase | ACHE |
| MOL000371 | Alpha-1B adrenergic receptor | ADRA1B |
| MOL000371 | Alpha-1D adrenergic receptor | ADRA1D |
| MOL000371 | Alpha-2C adrenergic receptor | ADRA2B |
| MOL000371 | Beta-1 adrenergic receptor | ADRB1R |
| MOL000371 | Beta-2 adrenergic receptor | ADRB2 |
| MOL000371 | Androgen receptor | AR |
| MOL000371 | Carbonic anhydrase II | CA2 |
| MOL000371 | Calmodulin | CALM1 |
| MOL000371 | Cyclin-A2 | CCNA2 |
| MOL000371 | Dipeptidyl peptidase IV | CD26 |
| MOL000371 | Cell division protein kinase 2 | Cdk2 |
| MOL000371 | Serine/threonine-protein kinase Chk1 | Chk1 |
| MOL000371 | Muscarinic acetylcholine receptor M1 | CHRM1 |
| MOL000371 | Muscarinic acetylcholine receptor M3 | Chrm3 |
| MOL000371 | Prostaglandin G/H synthase 1 | COX1 |
| MOL000371 | Estrogen receptor | ESR1 |
| MOL000371 | Estrogen receptor beta | ESR2 |
| MOL000371 | Thrombin | F2 |
| MOL000371 | Gamma-aminobutyric acid receptor subunit alpha-1 | GABRA1 |
| MOL000371 | Glycogen synthase kinase-3 beta | GSK3B |
| MOL000371 | 5-hydroxytryptamine receptor 3A | HTR3A |
| MOL000371 | Nitric oxide synthase, inducible | iNOS |
| MOL000371 | Mitogen-activated protein kinase 14 | MAPK14 |
| MOL000371 | Mu-type opioid receptor | MOR-1 |
| MOL000371 | Nuclear receptor coactivator 2 | NCOA2 |
| MOL000371 | Nitric-oxide synthase, endothelial | NOS |
| MOL000371 | CGMP-inhibited 3',5'-cyclic phosphodiesterase A | PDE3A |
| MOL000371 | Proto-oncogene serine/threonine-protein kinase Pim-1 | PIM1 |
| MOL000371 | Peroxisome proliferator activated receptor gamma | PPARG |
| MOL000371 | Trypsin-1 | PRSS1 |
| MOL000371 | Prostaglandin G/H synthase 2 | PTGS2 |
| MOL000371 | mRNA of Protein-tyrosine phosphatase, non-receptor type 1 | PTPN1 |
| MOL000371 | Retinoic acid receptor RXR-alpha | RXRA |
| MOL000371 | Sodium channel protein type 5 subunit alpha | SCN5A |
| MOL000378 | Alpha-1A adrenergic receptor | ADRA1A |
| MOL000378 | Alpha-1B adrenergic receptor | ADRA1B |
| MOL000378 | Alpha-1D adrenergic receptor | ADRA1D |
| MOL000378 | Alpha-2C adrenergic receptor | ADRA2B |
| MOL000378 | Beta-1 adrenergic receptor | ADRB1R |
| MOL000378 | Beta-2 adrenergic receptor | ADRB2 |
| MOL000378 | Androgen receptor | AR |
| MOL000378 | Carbonic anhydrase II | CA2 |
| MOL000378 | Calmodulin | CALM1 |
| MOL000378 | Cyclin-A2 | CCNA2 |
| MOL000378 | Dipeptidyl peptidase IV | CD26 |
| MOL000378 | Cell division protein kinase 2 | Cdk2 |
| MOL000378 | Serine/threonine-protein kinase Chk1 | Chk1 |
| MOL000378 | Muscarinic acetylcholine receptor M1 | CHRM1 |
| MOL000378 | Muscarinic acetylcholine receptor M2 | CHRM2 |
| MOL000378 | Muscarinic acetylcholine receptor M3 | Chrm3 |
| MOL000378 | Muscarinic acetylcholine receptor M4 | CHRM4 |
| MOL000378 | Muscarinic acetylcholine receptor M5 | CHRM5 |
| MOL000378 | Prostaglandin G/H synthase 1 | COX1 |
| MOL000378 | Dopamine D1 receptor | DRD1 |
| MOL000378 | Estrogen receptor | ESR1 |
| MOL000378 | Estrogen receptor beta | ESR2 |
| MOL000378 | Coagulation factor Xa | F10 |
| MOL000378 | Thrombin | F2 |
| MOL000378 | Gamma-aminobutyric acid receptor subunit alpha-1 | GABRA1 |
| MOL000378 | Glycogen synthase kinase-3 beta | GSK3B |
| MOL000378 | Heat shock protein HSP 90 | HSP90 |
| MOL000378 | 5-hydroxytryptamine 2A receptor | HTR2A |
| MOL000378 | Nitric oxide synthase, inducible | iNOS |
| MOL000378 | Potassium voltage-gated channel subfamily H member 2 | KCNH2 |
| MOL000378 | Calcium-activated potassium channel subunit alpha 1 | KCNMA1 |
| MOL000378 | Mitogen-activated protein kinase 14 | MAPK14 |
| MOL000378 | Nuclear receptor coactivator 2 | NCOA2 |
| MOL000378 | Nitric-oxide synthase, endothelial | NOS |
| MOL000378 | Delta-type opioid receptor | OPRD1 |
| MOL000378 | CGMP-inhibited 3',5'-cyclic phosphodiesterase A | PDE3A |
| MOL000378 | Proto-oncogene serine/threonine-protein kinase Pim-1 | PIM1 |
| MOL000378 | Peroxisome proliferator activated receptor gamma | PPARG |
| MOL000378 | mRNA of PKA Catalytic Subunit C-alpha | PRKACA |
| MOL000378 | Trypsin-1 | PRSS1 |
| MOL000378 | Prostaglandin G/H synthase 2 | PTGS2 |
| MOL000378 | mRNA of Protein-tyrosine phosphatase, non-receptor type 1 | PTPN1 |
| MOL000378 | Retinoic acid receptor RXR-alpha | RXRA |
| MOL000378 | Retinoic acid receptor RXR-beta | RXRB |
| MOL000378 | Sodium channel protein type 5 subunit alpha | SCN5A |
| MOL000378 | Sodium-dependent dopamine transporter | SLC6A3 |
| MOL000378 | Sodium-dependent serotonin transporter | SLC6A4 |
| MOL000379 | Carbonic anhydrase II | CA2 |
| MOL000379 | Cyclin-A2 | CCNA2 |
| MOL000379 | Thrombin | F2 |
| MOL000379 | Nuclear receptor coactivator 2 | NCOA2 |
| MOL000379 | Prostaglandin G/H synthase 2 | PTGS2 |
| MOL000379 | DNA topoisomerase II | TOP2 |
| MOL000380 | Acetylcholinesterase | ACHE |
| MOL000380 | Alpha-1B adrenergic receptor | ADRA1B |
| MOL000380 | Alpha-1D adrenergic receptor | ADRA1D |
| MOL000380 | Beta-2 adrenergic receptor | ADRB2 |
| MOL000380 | Androgen receptor | AR |
| MOL000380 | Carbonic anhydrase II | CA2 |
| MOL000380 | Calmodulin | CALM1 |
| MOL000380 | Cyclin-A2 | CCNA2 |
| MOL000380 | Dipeptidyl peptidase IV | CD26 |
| MOL000380 | Cell division protein kinase 2 | Cdk2 |
| MOL000380 | Serine/threonine-protein kinase Chk1 | Chk1 |
| MOL000380 | Muscarinic acetylcholine receptor M1 | CHRM1 |
| MOL000380 | Muscarinic acetylcholine receptor M3 | Chrm3 |
| MOL000380 | Muscarinic acetylcholine receptor M4 | CHRM4 |
| MOL000380 | Neuronal acetylcholine receptor protein, alpha-7 chain | CHRNA7 |
| MOL000380 | Prostaglandin G/H synthase 1 | COX1 |
| MOL000380 | Estrogen receptor | ESR1 |
| MOL000380 | Estrogen receptor beta | ESR2 |
| MOL000380 | Thrombin | F2 |
| MOL000380 | Gamma-aminobutyric acid receptor subunit alpha-1 | GABRA1 |
| MOL000380 | Glycogen synthase kinase-3 beta | GSK3B |
| MOL000380 | Heat shock protein HSP 90 | HSP90 |
| MOL000380 | 5-hydroxytryptamine receptor 3A | HTR3A |
| MOL000380 | Nitric oxide synthase, inducible | iNOS |
| MOL000380 | Mitogen-activated protein kinase 14 | MAPK14 |
| MOL000380 | Nuclear receptor coactivator 1 | NCOA1 |
| MOL000380 | Nuclear receptor coactivator 2 | NCOA2 |
| MOL000380 | Proto-oncogene serine/threonine-protein kinase Pim-1 | PIM1 |
| MOL000380 | Peroxisome proliferator activated receptor gamma | PPARG |
| MOL000380 | Trypsin-1 | PRSS1 |
| MOL000380 | Prostaglandin G/H synthase 2 | PTGS2 |
| MOL000380 | mRNA of Protein-tyrosine phosphatase, non-receptor type 1 | PTPN1 |
| MOL000380 | Retinoic acid receptor RXR-alpha | RXRA |
| MOL000380 | Sodium channel protein type 5 subunit alpha | SCN5A |
| MOL000387 | Androgen receptor | AR |
| MOL000387 | Dipeptidyl peptidase IV | CD26 |
| MOL000387 | Cell division protein kinase 2 | Cdk2 |
| MOL000387 | Prostaglandin G/H synthase 1 | COX1 |
| MOL000387 | Estrogen receptor | ESR1 |
| MOL000387 | Thrombin | F2 |
| MOL000387 | Glycogen synthase kinase-3 beta | GSK3B |
| MOL000387 | Heat shock protein HSP 90 | HSP90 |
| MOL000387 | Nitric oxide synthase, inducible | iNOS |
| MOL000387 | Calcium-activated potassium channel subunit alpha 1 | KCNMA1 |
| MOL000387 | Hepatocyte growth factor receptor | MET |
| MOL000387 | Prostaglandin G/H synthase 2 | PTGS2 |
| MOL000387 | DNA topoisomerase II | TOP2 |
| MOL000387 | Vascular endothelial growth factor receptor 2 | VEGFR2 |
| MOL000392 | Acetylcholinesterase | ACHE |
| MOL000392 | Alpha-1A adrenergic receptor | ADRA1A |
| MOL000392 | Beta-2 adrenergic receptor | ADRB2 |
| MOL000392 | Transcription factor AP-1 | AP1 |
| MOL000392 | Androgen receptor | AR |
| MOL000392 | ATP synthase subunit beta, mitochondrial | ATP5B precursor |
| MOL000392 | Carbonic anhydrase II | CA2 |
| MOL000392 | Calmodulin | CALM1 |
| MOL000392 | Cyclin-A2 | CCNA2 |
| MOL000392 | Dipeptidyl peptidase IV | CD26 |
| MOL000392 | Cell division protein kinase 2 | Cdk2 |
| MOL000392 | Serine/threonine-protein kinase Chk1 | Chk1 |
| MOL000392 | Muscarinic acetylcholine receptor M1 | CHRM1 |
| MOL000392 | Prostaglandin G/H synthase 1 | COX1 |
| MOL000392 | Estrogen receptor | ESR1 |
| MOL000392 | Estrogen receptor beta | ESR2 |
| MOL000392 | Thrombin | F2 |
| MOL000392 | Glycogen synthase kinase-3 beta | GSK3B |
| MOL000392 | Heat shock protein HSP 90 | HSP90 |
| MOL000392 | Nitric oxide synthase, inducible | iNOS |
| MOL000392 | Beta-lactamase | LACTB |
| MOL000392 | Amine oxidase [flavin-containing] B | MAOB |
| MOL000392 | Mitogen-activated protein kinase 14 | MAPK14 |
| MOL000392 | NADH-ubiquinone oxidoreductase chain 6 | MTND2 |
| MOL000392 | Nitric-oxide synthase, endothelial | NOS |
| MOL000392 | CGMP-inhibited 3',5'-cyclic phosphodiesterase A | PDE3A |
| MOL000392 | Proto-oncogene serine/threonine-protein kinase Pim-1 | PIM1 |
| MOL000392 | cAMP-dependent protein kinase inhibitor alpha | PKIA |
| MOL000392 | Peroxisome proliferator activated receptor gamma | PPARG |
| MOL000392 | mRNA of PKA Catalytic Subunit C-alpha | PRKACA |
| MOL000392 | Trypsin-1 | PRSS1 |
| MOL000392 | Prostaglandin G/H synthase 2 | PTGS2 |
| MOL000392 | mRNA of Protein-tyrosine phosphatase, non-receptor type 1 | PTPN1 |
| MOL000392 | Retinoic acid receptor RXR-alpha | RXRA |
| MOL000392 | Sodium-dependent dopamine transporter | SLC6A3 |
| MOL000392 | Sodium-dependent serotonin transporter | SLC6A4 |
| MOL000417 | Beta-2 adrenergic receptor | ADRB2 |
| MOL000417 | Androgen receptor | AR |
| MOL000417 | Carbonic anhydrase II | CA2 |
| MOL000417 | Calmodulin | CALM1 |
| MOL000417 | Cyclin-A2 | CCNA2 |
| MOL000417 | Dipeptidyl peptidase IV | CD26 |
| MOL000417 | Cell division protein kinase 2 | Cdk2 |
| MOL000417 | Serine/threonine-protein kinase Chk1 | Chk1 |
| MOL000417 | Prostaglandin G/H synthase 1 | COX1 |
| MOL000417 | Estrogen receptor | ESR1 |
| MOL000417 | Estrogen receptor beta | ESR2 |
| MOL000417 | Glycogen synthase kinase-3 beta | GSK3B |
| MOL000417 | Heat shock protein HSP 90 | HSP90 |
| MOL000417 | Nitric oxide synthase, inducible | iNOS |
| MOL000417 | Beta-lactamase | LACTB |
| MOL000417 | Mitogen-activated protein kinase 14 | MAPK14 |
| MOL000417 | Nuclear receptor coactivator 2 | NCOA2 |
| MOL000417 | CGMP-inhibited 3',5'-cyclic phosphodiesterase A | PDE3A |
| MOL000417 | Proto-oncogene serine/threonine-protein kinase Pim-1 | PIM1 |
| MOL000417 | Peroxisome proliferator activated receptor gamma | PPARG |
| MOL000417 | mRNA of PKA Catalytic Subunit C-alpha | PRKACA |
| MOL000417 | Trypsin-1 | PRSS1 |
| MOL000417 | Prostaglandin G/H synthase 2 | PTGS2 |
| MOL000417 | mRNA of Protein-tyrosine phosphatase, non-receptor type 1 | PTPN1 |
| MOL000417 | Retinoic acid receptor RXR-alpha | RXRA |
| MOL000422 | Acetylcholinesterase | ACHE |
| MOL000422 | Alpha-1B adrenergic receptor | ADRA1B |
| MOL000422 | Aryl hydrocarbon receptor | AHR |
| MOL000422 | Aldo-keto reductase family 1 member C3 | AKR1C3 |
| MOL000422 | Arachidonate 5-lipoxygenase | ALOX5 |
| MOL000422 | Transcription factor AP-1 | AP1 |
| MOL000422 | Androgen receptor | AR |
| MOL000422 | Apoptosis regulator Bcl-2 | BCL2 |
| MOL000422 | Carbonic anhydrase II | CA2 |
| MOL000422 | Calmodulin | CALM1 |
| MOL000422 | Cyclin-A2 | CCNA2 |
| MOL000422 | Dipeptidyl peptidase IV | CD26 |
| MOL000422 | Cell division protein kinase 2 | Cdk2 |
| MOL000422 | Serine/threonine-protein kinase Chk1 | Chk1 |
| MOL000422 | Muscarinic acetylcholine receptor M1 | CHRM1 |
| MOL000422 | Muscarinic acetylcholine receptor M2 | CHRM2 |
| MOL000422 | Prostaglandin G/H synthase 1 | COX1 |
| MOL000422 | Cell division control protein 2 homolog | CRK2 |
| MOL000422 | Cytochrome P450 1A2 | CYP1A2 |
| MOL000422 | Estrogen receptor | ESR1 |
| MOL000422 | Estrogen receptor beta | ESR2 |
| MOL000422 | Thrombin | F2 |
| MOL000422 | Coagulation factor VII | F7 |
| MOL000422 | Gamma-aminobutyric acid receptor subunit alpha-1 | GABRA1 |
| MOL000422 | Gamma-aminobutyric-acid receptor alpha-2 subunit | GABRA2 |
| MOL000422 | Glycogen synthase kinase-3 beta | GSK3B |
| MOL000422 | Glutathione S-transferase Mu 1 | GSTM1 |
| MOL000422 | Glutathione S-transferase Mu 2 | GSTM2 |
| MOL000422 | Glutathione S-transferase P | GSTP1 |
| MOL000422 | Heme oxygenase 1 | HMOX1 |
| MOL000422 | Heat shock protein HSP 90 | HSP90 |
| MOL000422 | Nitric oxide synthase, inducible | iNOS |
| MOL000422 | Insulin receptor | INSR |
| MOL000422 | Mitogen-activated protein kinase 8 | JNK1 |
| MOL000422 | Beta-lactamase | LACTB |
| MOL000422 | Mitogen-activated protein kinase 14 | MAPK14 |
| MOL000422 | Interstitial collagenase | MMP1 |
| MOL000422 | Sodium-dependent noradrenaline transporter | NAT1 |
| MOL000422 | Nuclear receptor coactivator 2 | NCOA2 |
| MOL000422 | Cytochrome P450 3A4 | NF-25 |
| MOL000422 | Nitric-oxide synthase, endothelial | NOS |
| MOL000422 | Progesterone receptor | PGR |
| MOL000422 | Phosphatidylinositol-4,5-bisphosphate 3-kinase catalytic subunit, gamma isoform | PIK3CG |
| MOL000422 | Proto-oncogene serine/threonine-protein kinase Pim-1 | PIM1 |
| MOL000422 | Peroxisome proliferator activated receptor gamma | PPARG |
| MOL000422 | Serine/threonine-protein phosphatase 2B catalytic subunit alpha isoform | PPP3CA |
| MOL000422 | mRNA of PKA Catalytic Subunit C-alpha | PRKACA |
| MOL000422 | Trypsin-1 | PRSS1 |
| MOL000422 | Prostaglandin G/H synthase 2 | PTGS2 |
| MOL000422 | mRNA of Protein-tyrosine phosphatase, non-receptor type 1 | PTPN1 |
| MOL000422 | E-selectin | SELE |
| MOL000422 | Tumor necrosis factor | TNF |
| MOL000422 | DNA topoisomerase II | TOP2 |
| MOL000422 | Vascular cell adhesion protein 1 | VCAM1 |
| MOL000422 | Xanthine dehydrogenase/oxidase | XD |
| MOL000433 | Cell division protein kinase 2 | Cdk2 |
| MOL000433 | Thrombin | F2 |
| MOL000433 | Glycogen synthase kinase-3 beta | GSK3B |
| MOL000439 | DNA topoisomerase II | TOP2 |
| MOL000442 | Androgen receptor | AR |
| MOL000442 | Cyclin-A2 | CCNA2 |
| MOL000442 | Cell division protein kinase 2 | Cdk2 |
| MOL000442 | Serine/threonine-protein kinase Chk1 | Chk1 |
| MOL000442 | Estrogen receptor | ESR1 |
| MOL000442 | Estrogen receptor beta | ESR2 |
| MOL000442 | Glycogen synthase kinase-3 beta | GSK3B |
| MOL000442 | Heat shock protein HSP 90 | HSP90 |
| MOL000442 | Nitric oxide synthase, inducible | iNOS |
| MOL000442 | Mitogen-activated protein kinase 14 | MAPK14 |
| MOL000442 | Proto-oncogene serine/threonine-protein kinase Pim-1 | PIM1 |
| MOL000442 | Peroxisome proliferator activated receptor gamma | PPARG |
| MOL000442 | Trypsin-1 | PRSS1 |
| MOL000442 | Prostaglandin G/H synthase 2 | PTGS2 |
| MOL000442 | mRNA of Protein-tyrosine phosphatase, non-receptor type 1 | PTPN1 |
| MOL000442 | Retinoic acid receptor RXR-alpha | RXRA |
| MOL000449 | Acetylcholinesterase | ACHE |
| MOL000449 | Alcohol dehydrogenase 1C | ADH1C |
| MOL000449 | Alpha-1A adrenergic receptor | ADRA1A |
| MOL000449 | Alpha-1B adrenergic receptor | ADRA1B |
| MOL000449 | Alpha-2A adrenergic receptor | ADRA2A |
| MOL000449 | Beta-1 adrenergic receptor | ADRB1R |
| MOL000449 | Beta-2 adrenergic receptor | ADRB2 |
| MOL000449 | Aldose reductase | ALDR1 |
| MOL000449 | Androgen receptor | AR |
| MOL000449 | Carbonic anhydrase II | CA2 |
| MOL000449 | Dipeptidyl peptidase IV | CD26 |
| MOL000449 | Cell division protein kinase 2 | Cdk2 |
| MOL000449 | Muscarinic acetylcholine receptor M1 | CHRM1 |
| MOL000449 | Muscarinic acetylcholine receptor M2 | CHRM2 |
| MOL000449 | Muscarinic acetylcholine receptor M3 | Chrm3 |
| MOL000449 | Neuronal acetylcholine receptor protein, alpha-7 chain | CHRNA7 |
| MOL000449 | Prostaglandin G/H synthase 1 | COX1 |
| MOL000449 | Chymotrypsinogen B | CTRB1 |
| MOL000449 | Estrogen receptor | ESR1 |
| MOL000449 | Thrombin | F2 |
| MOL000449 | Gamma-aminobutyric acid receptor subunit alpha-1 | GABRA1 |
| MOL000449 | Gamma-aminobutyric-acid receptor alpha-3 subunit | GABRA3 |
| MOL000449 | 5-hydroxytryptamine 2A receptor | HTR2A |
| MOL000449 | Ig gamma-1 chain C region | IGHG1 |
| MOL000449 | Nitric oxide synthase, inducible | iNOS |
| MOL000449 | Beta-lactamase | LACTB |
| MOL000449 | Leukotriene A-4 hydrolase | LTA4H |
| MOL000449 | Amine oxidase [flavin-containing] A | MAOA |
| MOL000449 | Amine oxidase [flavin-containing] B | MAOB |
| MOL000449 | Sodium-dependent noradrenaline transporter | NAT1 |
| MOL000449 | Nuclear receptor coactivator 1 | NCOA1 |
| MOL000449 | Nuclear receptor coactivator 2 | NCOA2 |
| MOL000449 | Nitric-oxide synthase, endothelial | NOS |
| MOL000449 | Glucocorticoid receptor | NR3C1 |
| MOL000449 | Mineralocorticoid receptor | NR3C2 |
| MOL000449 | Progesterone receptor | PGR |
| MOL000449 | Urokinase-type plasminogen activator | PLAU |
| MOL000449 | Peroxisome proliferator activated receptor gamma | PPARG |
| MOL000449 | mRNA of PKA Catalytic Subunit C-alpha | PRKACA |
| MOL000449 | Trypsin-1 | PRSS1 |
| MOL000449 | Prostaglandin G/H synthase 2 | PTGS2 |
| MOL000449 | Retinoic acid receptor RXR-alpha | RXRA |
| MOL000449 | Sodium channel protein type 5 subunit alpha | SCN5A |
| MOL000449 | Sodium-dependent dopamine transporter | SLC6A3 |
| MOL000519 | Acetylcholinesterase | ACHE |
| MOL000519 | Alpha-1B adrenergic receptor | ADRA1B |
| MOL000519 | Alpha-1D adrenergic receptor | ADRA1D |
| MOL000519 | Beta-2 adrenergic receptor | ADRB2 |
| MOL000519 | Androgen receptor | AR |
| MOL000519 | Carbonic anhydrase II | CA2 |
| MOL000519 | Cyclin-A2 | CCNA2 |
| MOL000519 | Dipeptidyl peptidase IV | CD26 |
| MOL000519 | Cell division protein kinase 2 | Cdk2 |
| MOL000519 | Muscarinic acetylcholine receptor M1 | CHRM1 |
| MOL000519 | Muscarinic acetylcholine receptor M3 | Chrm3 |
| MOL000519 | Neuronal acetylcholine receptor protein, alpha-7 chain | CHRNA7 |
| MOL000519 | Estrogen receptor | ESR1 |
| MOL000519 | Estrogen receptor beta | ESR2 |
| MOL000519 | Thrombin | F2 |
| MOL000519 | Glycogen synthase kinase-3 beta | GSK3B |
| MOL000519 | Ig gamma-1 chain C region | IGHG1 |
| MOL000519 | Nitric oxide synthase, inducible | iNOS |
| MOL000519 | Beta-lactamase | LACTB |
| MOL000519 | Mu-type opioid receptor | MOR-1 |
| MOL000519 | Nuclear receptor coactivator 1 | NCOA1 |
| MOL000519 | Nuclear receptor coactivator 2 | NCOA2 |
| MOL000519 | CGMP-inhibited 3',5'-cyclic phosphodiesterase A | PDE3A |
| MOL000519 | Proto-oncogene serine/threonine-protein kinase Pim-1 | PIM1 |
| MOL000519 | Peroxisome proliferator activated receptor gamma | PPARG |
| MOL000519 | Trypsin-1 | PRSS1 |
| MOL000519 | Prostaglandin G/H synthase 2 | PTGS2 |
| MOL000519 | Sodium channel protein type 5 subunit alpha | SCN5A |
| MOL000519 | DNA topoisomerase II | TOP2 |
| MOL000830 | Androgen receptor | AR |
| MOL000830 | Glucocorticoid receptor | NR3C1 |
| MOL000831 | Glucocorticoid receptor | NR3C1 |
| MOL000832 | Androgen receptor | AR |
| MOL000832 | Estrogen receptor | ESR1 |
| MOL000832 | Glucocorticoid receptor | NR3C1 |
| MOL000849 | Androgen receptor | AR |
| MOL000849 | Glucocorticoid receptor | NR3C1 |
| MOL000853 | Androgen receptor | AR |
| MOL000853 | Estrogen receptor | ESR1 |
| MOL000853 | Glucocorticoid receptor | NR3C1 |
| MOL000853 | Mineralocorticoid receptor | NR3C2 |
| MOL000854 | Androgen receptor | AR |
| MOL000854 | Glucocorticoid receptor | NR3C1 |
| MOL000856 | Androgen receptor | AR |
| MOL000856 | Glucocorticoid receptor | NR3C1 |
| MOL000862 | Glucocorticoid receptor | NR3C1 |
| MOL000953 | Androgen receptor | AR |
| MOL000953 | Dipeptidyl peptidase IV | CD26 |
| MOL000953 | Cytochrome P450-cam | CYP101A1 |
| MOL000953 | Estrogen receptor | ESR1 |
| MOL000953 | Nuclear receptor coactivator 2 | NCOA2 |
| MOL000953 | Glucocorticoid receptor | NR3C1 |
| MOL000953 | Mineralocorticoid receptor | NR3C2 |
| MOL000953 | Progesterone receptor | PGR |
| MOL000953 | Trypsin-1 | PRSS1 |
| MOL001006 | Androgen receptor | AR |
| MOL001006 | Estrogen receptor | ESR1 |
| MOL001006 | Nuclear receptor coactivator 2 | NCOA2 |
| MOL001006 | Glucocorticoid receptor | NR3C1 |
| MOL001006 | Mineralocorticoid receptor | NR3C2 |
| MOL001006 | Progesterone receptor | PGR |
| MOL001323 | Acetylcholinesterase | ACHE |
| MOL001323 | Alcohol dehydrogenase 1C | ADH1C |
| MOL001323 | Androgen receptor | AR |
| MOL001323 | Dipeptidyl peptidase IV | CD26 |
| MOL001323 | Cytochrome P450-cam | CYP101A1 |
| MOL001323 | Estrogen receptor | ESR1 |
| MOL001323 | Gamma-aminobutyric acid receptor subunit alpha-1 | GABRA1 |
| MOL001323 | Glucocorticoid receptor | NR3C1 |
| MOL001323 | Mineralocorticoid receptor | NR3C2 |
| MOL001323 | Progesterone receptor | PGR |
| MOL001323 | Trypsin-1 | PRSS1 |
| MOL001323 | Prostaglandin G/H synthase 2 | PTGS2 |
| MOL001494 | Acetylcholinesterase | ACHE |
| MOL001494 | Dipeptidyl peptidase IV | CD26 |
| MOL001494 | Prostaglandin G/H synthase 1 | COX1 |
| MOL001494 | Thrombin | F2 |
| MOL001494 | Nuclear receptor coactivator 2 | NCOA2 |
| MOL001494 | Nitric-oxide synthase, endothelial | NOS |
| MOL001494 | Peroxisome proliferator activated receptor gamma | PPARG |
| MOL001494 | Prostaglandin G/H synthase 2 | PTGS2 |
| MOL001755 | Androgen receptor | AR |
| MOL001755 | Estrogen receptor | ESR1 |
| MOL001755 | Glucocorticoid receptor | NR3C1 |
| MOL001755 | Mineralocorticoid receptor | NR3C2 |
| MOL001755 | Progesterone receptor | PGR |
| MOL001798 | Androgen receptor | AR |
| MOL001798 | Carbonic anhydrase II | CA2 |
| MOL001798 | Calmodulin | CALM1 |
| MOL001798 | Cyclin-A2 | CCNA2 |
| MOL001798 | Dipeptidyl peptidase IV | CD26 |
| MOL001798 | Cell division protein kinase 2 | Cdk2 |
| MOL001798 | Serine/threonine-protein kinase Chk1 | Chk1 |
| MOL001798 | Prostaglandin G/H synthase 1 | COX1 |
| MOL001798 | Estrogen receptor | ESR1 |
| MOL001798 | Estrogen receptor beta | ESR2 |
| MOL001798 | Glycogen synthase kinase-3 beta | GSK3B |
| MOL001798 | Heat shock protein HSP 90 | HSP90 |
| MOL001798 | Nitric oxide synthase, inducible | iNOS |
| MOL001798 | Beta-lactamase | LACTB |
| MOL001798 | Mitogen-activated protein kinase 14 | MAPK14 |
| MOL001798 | Phosphatidylinositol-4,5-bisphosphate 3-kinase catalytic subunit, gamma isoform | PIK3CG |
| MOL001798 | Proto-oncogene serine/threonine-protein kinase Pim-1 | PIM1 |
| MOL001798 | Peroxisome proliferator activated receptor gamma | PPARG |
| MOL001798 | mRNA of PKA Catalytic Subunit C-alpha | PRKACA |
| MOL001798 | Trypsin-1 | PRSS1 |
| MOL001798 | Prostaglandin G/H synthase 2 | PTGS2 |
| MOL001798 | mRNA of Protein-tyrosine phosphatase, non-receptor type 1 | PTPN1 |
| MOL001798 | Sodium channel protein type 5 subunit alpha | SCN5A |
| MOL001803 | Acetylcholinesterase | ACHE |
| MOL001803 | Alpha-1B adrenergic receptor | ADRA1B |
| MOL001803 | Beta-2 adrenergic receptor | ADRB2 |
| MOL001803 | Androgen receptor | AR |
| MOL001803 | Carbonic anhydrase II | CA2 |
| MOL001803 | Calmodulin | CALM1 |
| MOL001803 | Cyclin-A2 | CCNA2 |
| MOL001803 | Dipeptidyl peptidase IV | CD26 |
| MOL001803 | Serine/threonine-protein kinase Chk1 | Chk1 |
| MOL001803 | Prostaglandin G/H synthase 1 | COX1 |
| MOL001803 | Estrogen receptor | ESR1 |
| MOL001803 | Estrogen receptor beta | ESR2 |
| MOL001803 | Coagulation factor Xa | F10 |
| MOL001803 | Thrombin | F2 |
| MOL001803 | Coagulation factor VII | F7 |
| MOL001803 | Heat shock protein HSP 90 | HSP90 |
| MOL001803 | Nitric oxide synthase, inducible | iNOS |
| MOL001803 | Potassium voltage-gated channel subfamily H member 2 | KCNH2 |
| MOL001803 | Nuclear receptor coactivator 1 | NCOA1 |
| MOL001803 | Nuclear receptor coactivator 2 | NCOA2 |
| MOL001803 | Proto-oncogene serine/threonine-protein kinase Pim-1 | PIM1 |
| MOL001803 | Peroxisome proliferator activated receptor gamma | PPARG |
| MOL001803 | Trypsin-1 | PRSS1 |
| MOL001803 | Prostaglandin G/H synthase 2 | PTGS2 |
| MOL001803 | mRNA of Protein-tyrosine phosphatase, non-receptor type 1 | PTPN1 |
| MOL001803 | Sodium channel protein type 5 subunit alpha | SCN5A |
| MOL001803 | DNA topoisomerase II | TOP2 |
| MOL001941 | Androgen receptor | AR |
| MOL001941 | Cyclin-A2 | CCNA2 |
| MOL001941 | Dipeptidyl peptidase IV | CD26 |
| MOL001941 | Cell division protein kinase 2 | Cdk2 |
| MOL001941 | Serine/threonine-protein kinase Chk1 | Chk1 |
| MOL001941 | Muscarinic acetylcholine receptor M1 | CHRM1 |
| MOL001941 | Estrogen receptor | ESR1 |
| MOL001941 | Estrogen receptor beta | ESR2 |
| MOL001941 | Thrombin | F2 |
| MOL001941 | Gamma-aminobutyric acid receptor subunit alpha-1 | GABRA1 |
| MOL001941 | Glycogen synthase kinase-3 beta | GSK3B |
| MOL001941 | Nitric oxide synthase, inducible | iNOS |
| MOL001941 | Amine oxidase [flavin-containing] B | MAOB |
| MOL001941 | Mitogen-activated protein kinase 14 | MAPK14 |
| MOL001941 | Phosphatidylinositol-4,5-bisphosphate 3-kinase catalytic subunit, gamma isoform | PIK3CG |
| MOL001941 | Peroxisome proliferator activated receptor gamma | PPARG |
| MOL001941 | mRNA of PKA Catalytic Subunit C-alpha | PRKACA |
| MOL001941 | Prostaglandin G/H synthase 2 | PTGS2 |
| MOL002140 | Cyclin-A2 | CCNA2 |
| MOL002140 | Cell division protein kinase 2 | Cdk2 |
| MOL002140 | Serine/threonine-protein kinase Chk1 | Chk1 |
| MOL002140 | Estrogen receptor | ESR1 |
| MOL002140 | Estrogen receptor beta | ESR2 |
| MOL002140 | Thrombin | F2 |
| MOL002140 | Glycogen synthase kinase-3 beta | GSK3B |
| MOL002140 | Mitogen-activated protein kinase 14 | MAPK14 |
| MOL002140 | Proto-oncogene serine/threonine-protein kinase Pim-1 | PIM1 |
| MOL002140 | mRNA of PKA Catalytic Subunit C-alpha | PRKACA |
| MOL002140 | Prostaglandin G/H synthase 2 | PTGS2 |
| MOL002140 | Retinoic acid receptor RXR-alpha | RXRA |
| MOL002464 | Prostaglandin G/H synthase 1 | COX1 |
| MOL002464 | Thrombin | F2 |
| MOL002464 | Peroxisome proliferator activated receptor gamma | PPARG |
| MOL002670 | Acetylcholinesterase | ACHE |
| MOL002670 | Alpha-1B adrenergic receptor | ADRA1B |
| MOL002670 | Alpha-1D adrenergic receptor | ADRA1D |
| MOL002670 | Alpha-2C adrenergic receptor | ADRA2B |
| MOL002670 | Beta-1 adrenergic receptor | ADRB1R |
| MOL002670 | Beta-2 adrenergic receptor | ADRB2 |
| MOL002670 | Androgen receptor | AR |
| MOL002670 | Calmodulin | CALM1 |
| MOL002670 | Cyclin-A2 | CCNA2 |
| MOL002670 | Dipeptidyl peptidase IV | CD26 |
| MOL002670 | Cell division protein kinase 2 | Cdk2 |
| MOL002670 | Serine/threonine-protein kinase Chk1 | Chk1 |
| MOL002670 | Muscarinic acetylcholine receptor M1 | CHRM1 |
| MOL002670 | Muscarinic acetylcholine receptor M3 | Chrm3 |
| MOL002670 | Muscarinic acetylcholine receptor M4 | CHRM4 |
| MOL002670 | Muscarinic acetylcholine receptor M5 | CHRM5 |
| MOL002670 | Prostaglandin G/H synthase 1 | COX1 |
| MOL002670 | Dopamine D1 receptor | DRD1 |
| MOL002670 | Estrogen receptor | ESR1 |
| MOL002670 | Estrogen receptor beta | ESR2 |
| MOL002670 | Coagulation factor Xa | F10 |
| MOL002670 | Thrombin | F2 |
| MOL002670 | Coagulation factor VII | F7 |
| MOL002670 | Glycogen synthase kinase-3 beta | GSK3B |
| MOL002670 | Heat shock protein HSP 90 | HSP90 |
| MOL002670 | 5-hydroxytryptamine 2A receptor | HTR2A |
| MOL002670 | 5-hydroxytryptamine 2C receptor | HTR2C |
| MOL002670 | 5-hydroxytryptamine receptor 3A | HTR3A |
| MOL002670 | Nitric oxide synthase, inducible | iNOS |
| MOL002670 | Potassium voltage-gated channel subfamily H member 2 | KCNH2 |
| MOL002670 | Mitogen-activated protein kinase 14 | MAPK14 |
| MOL002670 | Mu-type opioid receptor | MOR-1 |
| MOL002670 | Nitric-oxide synthase, endothelial | NOS |
| MOL002670 | Delta-type opioid receptor | OPRD1 |
| MOL002670 | cAMP and cAMP-inhibited cGMP 3',5'-cyclic phosphodiesterase 10A | PDE10A |
| MOL002670 | Proto-oncogene serine/threonine-protein kinase Pim-1 | PIM1 |
| MOL002670 | Peroxisome proliferator activated receptor gamma | PPARG |
| MOL002670 | Trypsin-1 | PRSS1 |
| MOL002670 | Prostaglandin G/H synthase 2 | PTGS2 |
| MOL002670 | mRNA of Protein-tyrosine phosphatase, non-receptor type 1 | PTPN1 |
| MOL002670 | Retinoic acid receptor RXR-alpha | RXRA |
| MOL002670 | Retinoic acid receptor RXR-beta | RXRB |
| MOL002670 | Sodium channel protein type 5 subunit alpha | SCN5A |
| MOL002670 | Sodium-dependent serotonin transporter | SLC6A4 |
| MOL002670 | DNA topoisomerase II | TOP2 |
| MOL002714 | Aryl hydrocarbon receptor | AHR |
| MOL002714 | Androgen receptor | AR |
| MOL002714 | Apoptosis regulator Bcl-2 | BCL2 |
| MOL002714 | Carbonic anhydrase II | CA2 |
| MOL002714 | Calmodulin | CALM1 |
| MOL002714 | Cyclin-A2 | CCNA2 |
| MOL002714 | Dipeptidyl peptidase IV | CD26 |
| MOL002714 | Cell division protein kinase 2 | Cdk2 |
| MOL002714 | Serine/threonine-protein kinase Chk1 | Chk1 |
| MOL002714 | Prostaglandin G/H synthase 1 | COX1 |
| MOL002714 | Cell division control protein 2 homolog | CRK2 |
| MOL002714 | Cytochrome c | CYCS |
| MOL002714 | Estrogen receptor | ESR1 |
| MOL002714 | Estrogen receptor beta | ESR2 |
| MOL002714 | Glycogen synthase kinase-3 beta | GSK3B |
| MOL002714 | Heat shock protein HSP 90 | HSP90 |
| MOL002714 | Nitric oxide synthase, inducible | iNOS |
| MOL002714 | Beta-lactamase | LACTB |
| MOL002714 | Mitogen-activated protein kinase 14 | MAPK14 |
| MOL002714 | Myeloperoxidase | MPO |
| MOL002714 | Nuclear receptor coactivator 1 | NCOA1 |
| MOL002714 | Nuclear receptor coactivator 2 | NCOA2 |
| MOL002714 | Cellular tumor antigen p53 | P53_HUMAN |
| MOL002714 | CGMP-inhibited 3',5'-cyclic phosphodiesterase A | PDE3A |
| MOL002714 | Egl nine homolog 1 | PHD2 |
| MOL002714 | Phosphatidylinositol-4,5-bisphosphate 3-kinase catalytic subunit, gamma isoform | PIK3CG |
| MOL002714 | Proto-oncogene serine/threonine-protein kinase Pim-1 | PIM1 |
| MOL002714 | Peroxisome proliferator activated receptor gamma | PPARG |
| MOL002714 | mRNA of PKA Catalytic Subunit C-alpha | PRKACA |
| MOL002714 | Trypsin-1 | PRSS1 |
| MOL002714 | Prostaglandin G/H synthase 2 | PTGS2 |
| MOL002714 | mRNA of Protein-tyrosine phosphatase, non-receptor type 1 | PTPN1 |
| MOL002714 | Vascular endothelial growth factor A | VEGFA |
| MOL002776 | Carbonic anhydrase II | CA2 |
| MOL002776 | Cyclin-A2 | CCNA2 |
| MOL002776 | Coagulation factor Xa | F10 |
| MOL002776 | Glycogen synthase kinase-3 beta | GSK3B |
| MOL002776 | Proto-oncogene serine/threonine-protein kinase Pim-1 | PIM1 |
| MOL002776 | mRNA of Protein-tyrosine phosphatase, non-receptor type 1 | PTPN1 |
| MOL002879 | Beta-2 adrenergic receptor | ADRB2 |
| MOL002879 | Dipeptidyl peptidase IV | CD26 |
| MOL002879 | Muscarinic acetylcholine receptor M3 | Chrm3 |
| MOL002879 | Estrogen receptor | ESR1 |
| MOL002879 | Thrombin | F2 |
| MOL002879 | Peroxisome proliferator activated receptor gamma | PPARG |
| MOL002879 | Trypsin-1 | PRSS1 |
| MOL002879 | Sodium channel protein type 5 subunit alpha | SCN5A |
| MOL002882 | Thrombin | F2 |
| MOL002882 | Nitric-oxide synthase, endothelial | NOS |
| MOL002882 | Peroxisome proliferator activated receptor gamma | PPARG |
| MOL002914 | Androgen receptor | AR |
| MOL002914 | Carbonic anhydrase II | CA2 |
| MOL002914 | Calmodulin | CALM1 |
| MOL002914 | Cyclin-A2 | CCNA2 |
| MOL002914 | Dipeptidyl peptidase IV | CD26 |
| MOL002914 | Cell division protein kinase 2 | Cdk2 |
| MOL002914 | Prostaglandin G/H synthase 1 | COX1 |
| MOL002914 | Estrogen receptor | ESR1 |
| MOL002914 | Estrogen receptor beta | ESR2 |
| MOL002914 | Glycogen synthase kinase-3 beta | GSK3B |
| MOL002914 | Heat shock protein HSP 90 | HSP90 |
| MOL002914 | Nitric oxide synthase, inducible | iNOS |
| MOL002914 | Beta-lactamase | LACTB |
| MOL002914 | Mitogen-activated protein kinase 14 | MAPK14 |
| MOL002914 | Nuclear receptor coactivator 2 | NCOA2 |
| MOL002914 | Phosphatidylinositol-4,5-bisphosphate 3-kinase catalytic subunit, gamma isoform | PIK3CG |
| MOL002914 | Proto-oncogene serine/threonine-protein kinase Pim-1 | PIM1 |
| MOL002914 | Peroxisome proliferator activated receptor gamma | PPARG |
| MOL002914 | mRNA of PKA Catalytic Subunit C-alpha | PRKACA |
| MOL002914 | Trypsin-1 | PRSS1 |
| MOL002914 | Prostaglandin G/H synthase 2 | PTGS2 |
| MOL002914 | mRNA of Protein-tyrosine phosphatase, non-receptor type 1 | PTPN1 |
| MOL002914 | Glycogen phosphorylase, muscle form | PYGM |
| MOL003036 | Androgen receptor | AR |
| MOL003036 | Estrogen receptor | ESR1 |
| MOL003036 | Nuclear receptor coactivator 2 | NCOA2 |
| MOL003036 | Glucocorticoid receptor | NR3C1 |
| MOL003036 | Mineralocorticoid receptor | NR3C2 |
| MOL003036 | Progesterone receptor | PGR |
| MOL003578 | Androgen receptor | AR |
| MOL003578 | Estrogen receptor | ESR1 |
| MOL003578 | Mineralocorticoid receptor | NR3C2 |
| MOL003896 | Acetylcholinesterase | ACHE |
| MOL003896 | Alpha-1B adrenergic receptor | ADRA1B |
| MOL003896 | Alpha-1D adrenergic receptor | ADRA1D |
| MOL003896 | Beta-1 adrenergic receptor | ADRB1R |
| MOL003896 | Beta-2 adrenergic receptor | ADRB2 |
| MOL003896 | Androgen receptor | AR |
| MOL003896 | Carbonic anhydrase II | CA2 |
| MOL003896 | Calmodulin | CALM1 |
| MOL003896 | Cyclin-A2 | CCNA2 |
| MOL003896 | Dipeptidyl peptidase IV | CD26 |
| MOL003896 | Cell division protein kinase 2 | Cdk2 |
| MOL003896 | Serine/threonine-protein kinase Chk1 | Chk1 |
| MOL003896 | Muscarinic acetylcholine receptor M1 | CHRM1 |
| MOL003896 | Muscarinic acetylcholine receptor M3 | Chrm3 |
| MOL003896 | Muscarinic acetylcholine receptor M5 | CHRM5 |
| MOL003896 | Neuronal acetylcholine receptor protein, alpha-7 chain | CHRNA7 |
| MOL003896 | Prostaglandin G/H synthase 1 | COX1 |
| MOL003896 | Dopamine D1 receptor | DRD1 |
| MOL003896 | Estrogen receptor | ESR1 |
| MOL003896 | Estrogen receptor beta | ESR2 |
| MOL003896 | Thrombin | F2 |
| MOL003896 | Gamma-aminobutyric acid receptor subunit alpha-1 | GABRA1 |
| MOL003896 | Glycogen synthase kinase-3 beta | GSK3B |
| MOL003896 | Heat shock protein HSP 90 | HSP90 |
| MOL003896 | Ig gamma-1 chain C region | IGHG1 |
| MOL003896 | Nitric oxide synthase, inducible | iNOS |
| MOL003896 | Leukotriene A-4 hydrolase | LTA4H |
| MOL003896 | Amine oxidase [flavin-containing] B | MAOB |
| MOL003896 | Mitogen-activated protein kinase 14 | MAPK14 |
| MOL003896 | Mu-type opioid receptor | MOR-1 |
| MOL003896 | Nuclear receptor coactivator 1 | NCOA1 |
| MOL003896 | Nuclear receptor coactivator 2 | NCOA2 |
| MOL003896 | Nitric-oxide synthase, endothelial | NOS |
| MOL003896 | CGMP-inhibited 3',5'-cyclic phosphodiesterase A | PDE3A |
| MOL003896 | Proto-oncogene serine/threonine-protein kinase Pim-1 | PIM1 |
| MOL003896 | cAMP-dependent protein kinase inhibitor alpha | PKIA |
| MOL003896 | Peroxisome proliferator activated receptor gamma | PPARG |
| MOL003896 | mRNA of PKA Catalytic Subunit C-alpha | PRKACA |
| MOL003896 | Trypsin-1 | PRSS1 |
| MOL003896 | Prostaglandin G/H synthase 2 | PTGS2 |
| MOL003896 | mRNA of Protein-tyrosine phosphatase, non-receptor type 1 | PTPN1 |
| MOL003896 | Retinoic acid receptor RXR-alpha | RXRA |
| MOL003896 | Sodium channel protein type 5 subunit alpha | SCN5A |
| MOL003896 | Sodium-dependent dopamine transporter | SLC6A3 |
| MOL003896 | Sodium-dependent serotonin transporter | SLC6A4 |
| MOL004328 | 4-aminobutyrate aminotransferase, mitochondrial | ABAT |
| MOL004328 | Multidrug resistance-associated protein 1 | ABCC1 |
| MOL004328 | Aldo-keto reductase family 1 member C1 | AKR1C1 |
| MOL004328 | Androgen receptor | AR |
| MOL004328 | Apoptosis regulator Bcl-2 | BCL2 |
| MOL004328 | Carbonic anhydrase II | CA2 |
| MOL004328 | Catalase | CAT |
| MOL004328 | Cyclin-A2 | CCNA2 |
| MOL004328 | Dipeptidyl peptidase IV | CD26 |
| MOL004328 | Cell division protein kinase 2 | Cdk2 |
| MOL004328 | Prostaglandin G/H synthase 1 | COX1 |
| MOL004328 | Mitogen-activated protein kinase 3 | ERK1 |
| MOL004328 | Mitogen-activated protein kinase 1 | ERK2 |
| MOL004328 | Estrogen receptor | ESR1 |
| MOL004328 | Estrogen receptor beta | ESR2 |
| MOL004328 | Fatty acid synthase | FAS |
| MOL004328 | Aspartate aminotransferase, cytoplasmic | GOT1 |
| MOL004328 | Glycogen synthase kinase-3 beta | GSK3B |
| MOL004328 | Glutathione reductase, mitochondrial | GSR |
| MOL004328 | Glutathione S-transferase P | GSTP1 |
| MOL004328 | 3-hydroxy-3-methylglutaryl-coenzyme A reductase | HMGCR |
| MOL004328 | Heat shock protein HSP 90 | HSP90 |
| MOL004328 | Nitric oxide synthase, inducible | iNOS |
| MOL004328 | Beta-lactamase | LACTB |
| MOL004328 | Low-density lipoprotein receptor | LDLR |
| MOL004328 | Mitogen-activated protein kinase 14 | MAPK14 |
| MOL004328 | Phosphatidylinositol-4,5-bisphosphate 3-kinase catalytic subunit, gamma isoform | PIK3CG |
| MOL004328 | Peroxisome proliferator activated receptor gamma | PPARG |
| MOL004328 | mRNA of PKA Catalytic Subunit C-alpha | PRKACA |
| MOL004328 | Prostaglandin G/H synthase 2 | PTGS2 |
| MOL004328 | mRNA of Protein-tyrosine phosphatase, non-receptor type 1 | PTPN1 |
| MOL004328 | Sterol O-acyltransferase 1 | SOAT1 |
| MOL004328 | Superoxide dismutase [Cu-Zn] | SOD |
| MOL004355 | Androgen receptor | AR |
| MOL004355 | Estrogen receptor | ESR1 |
| MOL004355 | Nuclear receptor coactivator 2 | NCOA2 |
| MOL004355 | Glucocorticoid receptor | NR3C1 |
| MOL004355 | Mineralocorticoid receptor | NR3C2 |
| MOL004355 | Progesterone receptor | PGR |
| MOL004492 | Thrombin | F2 |
| MOL005030 | Acetylcholinesterase | ACHE |
| MOL005030 | Prostaglandin G/H synthase 1 | COX1 |
| MOL005030 | Thrombin | F2 |
| MOL005030 | Nuclear receptor coactivator 2 | NCOA2 |
| MOL005030 | Nitric-oxide synthase, endothelial | NOS |
| MOL005030 | Peroxisome proliferator activated receptor gamma | PPARG |
| MOL005100 | Androgen receptor | AR |
| MOL005100 | Carbonic anhydrase II | CA2 |
| MOL005100 | Calmodulin | CALM1 |
| MOL005100 | Cyclin-A2 | CCNA2 |
| MOL005100 | Dipeptidyl peptidase IV | CD26 |
| MOL005100 | Cell division protein kinase 2 | Cdk2 |
| MOL005100 | Serine/threonine-protein kinase Chk1 | Chk1 |
| MOL005100 | Prostaglandin G/H synthase 1 | COX1 |
| MOL005100 | Estrogen receptor | ESR1 |
| MOL005100 | Estrogen receptor beta | ESR2 |
| MOL005100 | Glycogen synthase kinase-3 beta | GSK3B |
| MOL005100 | Heat shock protein HSP 90 | HSP90 |
| MOL005100 | Nitric oxide synthase, inducible | iNOS |
| MOL005100 | Beta-lactamase | LACTB |
| MOL005100 | Mitogen-activated protein kinase 14 | MAPK14 |
| MOL005100 | Nuclear receptor coactivator 1 | NCOA1 |
| MOL005100 | Nuclear receptor coactivator 2 | NCOA2 |
| MOL005100 | Phosphatidylinositol-4,5-bisphosphate 3-kinase catalytic subunit, gamma isoform | PIK3CG |
| MOL005100 | Proto-oncogene serine/threonine-protein kinase Pim-1 | PIM1 |
| MOL005100 | Peroxisome proliferator activated receptor gamma | PPARG |
| MOL005100 | mRNA of PKA Catalytic Subunit C-alpha | PRKACA |
| MOL005100 | Trypsin-1 | PRSS1 |
| MOL005100 | Prostaglandin G/H synthase 2 | PTGS2 |
| MOL005100 | mRNA of Protein-tyrosine phosphatase, non-receptor type 1 | PTPN1 |
| MOL005100 | Sodium channel protein type 5 subunit alpha | SCN5A |
| MOL005100 | DNA topoisomerase II | TOP2 |
| MOL005321 | Acetylcholinesterase | ACHE |
| MOL005321 | Beta-2 adrenergic receptor | ADRB2 |
| MOL005321 | Androgen receptor | AR |
| MOL005321 | Carbonic anhydrase II | CA2 |
| MOL005321 | Cyclin-A2 | CCNA2 |
| MOL005321 | Dipeptidyl peptidase IV | CD26 |
| MOL005321 | Cell division protein kinase 2 | Cdk2 |
| MOL005321 | Serine/threonine-protein kinase Chk1 | Chk1 |
| MOL005321 | Neuronal acetylcholine receptor protein, alpha-7 chain | CHRNA7 |
| MOL005321 | Prostaglandin G/H synthase 1 | COX1 |
| MOL005321 | Estrogen receptor | ESR1 |
| MOL005321 | Estrogen receptor beta | ESR2 |
| MOL005321 | Thrombin | F2 |
| MOL005321 | Gamma-aminobutyric acid receptor subunit alpha-1 | GABRA1 |
| MOL005321 | Glycogen synthase kinase-3 beta | GSK3B |
| MOL005321 | Heat shock protein HSP 90 | HSP90 |
| MOL005321 | Nitric oxide synthase, inducible | iNOS |
| MOL005321 | Mitogen-activated protein kinase 14 | MAPK14 |
| MOL005321 | CGMP-inhibited 3',5'-cyclic phosphodiesterase A | PDE3A |
| MOL005321 | Phosphatidylinositol-4,5-bisphosphate 3-kinase catalytic subunit, gamma isoform | PIK3CG |
| MOL005321 | Proto-oncogene serine/threonine-protein kinase Pim-1 | PIM1 |
| MOL005321 | Peroxisome proliferator activated receptor gamma | PPARG |
| MOL005321 | mRNA of PKA Catalytic Subunit C-alpha | PRKACA |
| MOL005321 | Prostaglandin G/H synthase 2 | PTGS2 |
| MOL005321 | mRNA of Protein-tyrosine phosphatase, non-receptor type 1 | PTPN1 |
| MOL005321 | Retinoic acid receptor RXR-alpha | RXRA |
| MOL005321 | Sodium channel protein type 5 subunit alpha | SCN5A |
| MOL005815 | Acetylcholinesterase | ACHE |
| MOL005815 | Androgen receptor | AR |
| MOL005815 | Carbonic anhydrase II | CA2 |
| MOL005815 | Calmodulin | CALM1 |
| MOL005815 | Dipeptidyl peptidase IV | CD26 |
| MOL005815 | Estrogen receptor | ESR1 |
| MOL005815 | Estrogen receptor beta | ESR2 |
| MOL005815 | Coagulation factor Xa | F10 |
| MOL005815 | Thrombin | F2 |
| MOL005815 | Coagulation factor VII | F7 |
| MOL005815 | Glycogen synthase kinase-3 beta | GSK3B |
| MOL005815 | Heat shock protein HSP 90 | HSP90 |
| MOL005815 | Nitric oxide synthase, inducible | iNOS |
| MOL005815 | Potassium voltage-gated channel subfamily H member 2 | KCNH2 |
| MOL005815 | Calcium-activated potassium channel subunit alpha 1 | KCNMA1 |
| MOL005815 | Nuclear receptor coactivator 2 | NCOA2 |
| MOL005815 | Proto-oncogene serine/threonine-protein kinase Pim-1 | PIM1 |
| MOL005815 | Peroxisome proliferator activated receptor gamma | PPARG |
| MOL005815 | Trypsin-1 | PRSS1 |
| MOL005815 | Prostaglandin G/H synthase 2 | PTGS2 |
| MOL005815 | mRNA of Protein-tyrosine phosphatase, non-receptor type 1 | PTPN1 |
| MOL005815 | Sodium channel protein type 5 subunit alpha | SCN5A |
| MOL005815 | DNA topoisomerase II | TOP2 |
| MOL005828 | Transcription factor AP-1 | AP1 |
| MOL005828 | Androgen receptor | AR |
| MOL005828 | Apoptosis regulator Bcl-2 | BCL2 |
| MOL005828 | Carbonic anhydrase II | CA2 |
| MOL005828 | Calmodulin | CALM1 |
| MOL005828 | Cyclin-A2 | CCNA2 |
| MOL005828 | Dipeptidyl peptidase IV | CD26 |
| MOL005828 | Serine/threonine-protein kinase Chk1 | Chk1 |
| MOL005828 | Prostaglandin G/H synthase 1 | COX1 |
| MOL005828 | Cytosolic phospholipase A2 | cPLA2 |
| MOL005828 | Estrogen receptor | ESR1 |
| MOL005828 | Estrogen receptor beta | ESR2 |
| MOL005828 | Coagulation factor Xa | F10 |
| MOL005828 | Thrombin | F2 |
| MOL005828 | Coagulation factor VII | F7 |
| MOL005828 | Glycogen synthase kinase-3 beta | GSK3B |
| MOL005828 | Heat shock protein HSP 90 | HSP90 |
| MOL005828 | Nitric oxide synthase, inducible | iNOS |
| MOL005828 | Mitogen-activated protein kinase 8 | JNK1 |
| MOL005828 | Potassium voltage-gated channel subfamily H member 2 | KCNH2 |
| MOL005828 | Calcium-activated potassium channel subunit alpha 1 | KCNMA1 |
| MOL005828 | Nuclear receptor coactivator 2 | NCOA2 |
| MOL005828 | Cellular tumor antigen p53 | P53_HUMAN |
| MOL005828 | Proto-oncogene serine/threonine-protein kinase Pim-1 | PIM1 |
| MOL005828 | Peroxisome proliferator activated receptor gamma | PPARG |
| MOL005828 | Trypsin-1 | PRSS1 |
| MOL005828 | Prostaglandin G/H synthase 2 | PTGS2 |
| MOL005828 | mRNA of Protein-tyrosine phosphatase, non-receptor type 1 | PTPN1 |
| MOL005828 | Sodium channel protein type 5 subunit alpha | SCN5A |
| MOL005828 | DNA topoisomerase II | TOP2 |
| MOL005849 | Beta-2 adrenergic receptor | ADRB2 |
| MOL005849 | Androgen receptor | AR |
| MOL005849 | Carbonic anhydrase II | CA2 |
| MOL005849 | Calmodulin | CALM1 |
| MOL005849 | Cyclin-A2 | CCNA2 |
| MOL005849 | Dipeptidyl peptidase IV | CD26 |
| MOL005849 | Cell division protein kinase 2 | Cdk2 |
| MOL005849 | Prostaglandin G/H synthase 1 | COX1 |
| MOL005849 | Estrogen receptor | ESR1 |
| MOL005849 | Estrogen receptor beta | ESR2 |
| MOL005849 | Glycogen synthase kinase-3 beta | GSK3B |
| MOL005849 | Heat shock protein HSP 90 | HSP90 |
| MOL005849 | Nitric oxide synthase, inducible | iNOS |
| MOL005849 | Beta-lactamase | LACTB |
| MOL005849 | Mitogen-activated protein kinase 14 | MAPK14 |
| MOL005849 | Nuclear receptor coactivator 1 | NCOA1 |
| MOL005849 | Nuclear receptor coactivator 2 | NCOA2 |
| MOL005849 | Phosphatidylinositol-4,5-bisphosphate 3-kinase catalytic subunit, gamma isoform | PIK3CG |
| MOL005849 | Proto-oncogene serine/threonine-protein kinase Pim-1 | PIM1 |
| MOL005849 | Peroxisome proliferator activated receptor gamma | PPARG |
| MOL005849 | mRNA of PKA Catalytic Subunit C-alpha | PRKACA |
| MOL005849 | Trypsin-1 | PRSS1 |
| MOL005849 | Prostaglandin G/H synthase 2 | PTGS2 |
| MOL005849 | mRNA of Protein-tyrosine phosphatase, non-receptor type 1 | PTPN1 |
| MOL005849 | Retinoic acid receptor RXR-alpha | RXRA |
| MOL005849 | Sodium channel protein type 5 subunit alpha | SCN5A |
| MOL006554 | Androgen receptor | AR |
| MOL006554 | Estrogen receptor | ESR1 |
| MOL006774 | Androgen receptor | AR |
| MOL006774 | Estrogen receptor | ESR1 |
| MOL006774 | Nuclear receptor coactivator 2 | NCOA2 |
| MOL006774 | Progesterone receptor | PGR |
| MOL006936 | Acetylcholinesterase | ACHE |
| MOL006936 | Carbonic anhydrase II | CA2 |
| MOL006936 | Prostaglandin G/H synthase 1 | COX1 |
| MOL006936 | Thrombin | F2 |
| MOL006936 | Nitric oxide synthase, inducible | iNOS |
| MOL006936 | Nuclear receptor coactivator 2 | NCOA2 |
| MOL006936 | Nitric-oxide synthase, endothelial | NOS |
| MOL006936 | Peroxisome proliferator activated receptor gamma | PPARG |
| MOL006937 | Estrogen receptor | ESR1 |
| MOL006937 | Thrombin | F2 |
| MOL006937 | Nitric-oxide synthase, endothelial | NOS |
| MOL006937 | Peroxisome proliferator activated receptor gamma | PPARG |
| MOL006957 | Beta-2 adrenergic receptor | ADRB2 |
| MOL006957 | Androgen receptor | AR |
| MOL006957 | Carbonic anhydrase II | CA2 |
| MOL006957 | Calmodulin | CALM1 |
| MOL006957 | Cyclin-A2 | CCNA2 |
| MOL006957 | Dipeptidyl peptidase IV | CD26 |
| MOL006957 | Cell division protein kinase 2 | Cdk2 |
| MOL006957 | Serine/threonine-protein kinase Chk1 | Chk1 |
| MOL006957 | Estrogen receptor | ESR1 |
| MOL006957 | Thrombin | F2 |
| MOL006957 | Glycogen synthase kinase-3 beta | GSK3B |
| MOL006957 | Nitric oxide synthase, inducible | iNOS |
| MOL006957 | Mitogen-activated protein kinase 14 | MAPK14 |
| MOL006957 | Peroxisome proliferator activated receptor gamma | PPARG |
| MOL006957 | Trypsin-1 | PRSS1 |
| MOL006957 | Prostaglandin G/H synthase 2 | PTGS2 |
| MOL006967 | Cyclin-A2 | CCNA2 |
| MOL006967 | Dipeptidyl peptidase IV | CD26 |
| MOL006967 | Cell division protein kinase 2 | Cdk2 |
| MOL006967 | Estrogen receptor | ESR1 |
| MOL006967 | Thrombin | F2 |
| MOL006967 | Nitric oxide synthase, inducible | iNOS |
| MOL006967 | Purine nucleoside phosphorylase | PNP |
| MOL006967 | Prostaglandin G/H synthase 2 | PTGS2 |
| MOL007059 | Acetylcholinesterase | ACHE |
| MOL007059 | Alpha-1A adrenergic receptor | ADRA1A |
| MOL007059 | Beta-2 adrenergic receptor | ADRB2 |
| MOL007059 | Androgen receptor | AR |
| MOL007059 | Carbonic anhydrase II | CA2 |
| MOL007059 | Cyclin-A2 | CCNA2 |
| MOL007059 | Dipeptidyl peptidase IV | CD26 |
| MOL007059 | Cell division protein kinase 2 | Cdk2 |
| MOL007059 | Serine/threonine-protein kinase Chk1 | Chk1 |
| MOL007059 | Muscarinic acetylcholine receptor M1 | CHRM1 |
| MOL007059 | Neuronal acetylcholine receptor protein, alpha-7 chain | CHRNA7 |
| MOL007059 | Dopamine D1 receptor | DRD1 |
| MOL007059 | Estrogen receptor | ESR1 |
| MOL007059 | Estrogen receptor beta | ESR2 |
| MOL007059 | Thrombin | F2 |
| MOL007059 | Glycogen synthase kinase-3 beta | GSK3B |
| MOL007059 | Heat shock protein HSP 90 | HSP90 |
| MOL007059 | Ig gamma-1 chain C region | IGHG1 |
| MOL007059 | Nitric oxide synthase, inducible | iNOS |
| MOL007059 | Mitogen-activated protein kinase 14 | MAPK14 |
| MOL007059 | Mu-type opioid receptor | MOR-1 |
| MOL007059 | Nuclear receptor coactivator 1 | NCOA1 |
| MOL007059 | Nitric-oxide synthase, endothelial | NOS |
| MOL007059 | Delta-type opioid receptor | OPRD1 |
| MOL007059 | Proto-oncogene serine/threonine-protein kinase Pim-1 | PIM1 |
| MOL007059 | Peroxisome proliferator activated receptor gamma | PPARG |
| MOL007059 | Trypsin-1 | PRSS1 |
| MOL007059 | Prostaglandin G/H synthase 2 | PTGS2 |
| MOL007059 | mRNA of Protein-tyrosine phosphatase, non-receptor type 1 | PTPN1 |
| MOL007059 | Retinoic acid receptor RXR-alpha | RXRA |
| MOL007206 | Acetylcholinesterase | ACHE |
| MOL007206 | Alpha-1B adrenergic receptor | ADRA1B |
| MOL007206 | Alpha-1D adrenergic receptor | ADRA1D |
| MOL007206 | Alpha-2C adrenergic receptor | ADRA2B |
| MOL007206 | Alpha-2B adrenergic receptor | ADRA2B |
| MOL007206 | Beta-1 adrenergic receptor | ADRB1R |
| MOL007206 | Beta-2 adrenergic receptor | ADRB2 |
| MOL007206 | Androgen receptor | AR |
| MOL007206 | Calmodulin | CALM1 |
| MOL007206 | Cyclin-A2 | CCNA2 |
| MOL007206 | Dipeptidyl peptidase IV | CD26 |
| MOL007206 | Cell division protein kinase 2 | Cdk2 |
| MOL007206 | Serine/threonine-protein kinase Chk1 | Chk1 |
| MOL007206 | Muscarinic acetylcholine receptor M1 | CHRM1 |
| MOL007206 | Muscarinic acetylcholine receptor M2 | CHRM2 |
| MOL007206 | Muscarinic acetylcholine receptor M3 | Chrm3 |
| MOL007206 | Muscarinic acetylcholine receptor M4 | CHRM4 |
| MOL007206 | Muscarinic acetylcholine receptor M5 | CHRM5 |
| MOL007206 | Prostaglandin G/H synthase 1 | COX1 |
| MOL007206 | D(3) dopamine receptor | DRD3 |
| MOL007206 | Estrogen receptor | ESR1 |
| MOL007206 | Estrogen receptor beta | ESR2 |
| MOL007206 | Thrombin | F2 |
| MOL007206 | Glycogen synthase kinase-3 beta | GSK3B |
| MOL007206 | Heat shock protein HSP 90 | HSP90 |
| MOL007206 | 5-hydroxytryptamine 1B receptor | HTR1B |
| MOL007206 | 5-hydroxytryptamine 2A receptor | HTR2A |
| MOL007206 | 5-hydroxytryptamine 2C receptor | HTR2C |
| MOL007206 | Nitric oxide synthase, inducible | iNOS |
| MOL007206 | Potassium voltage-gated channel subfamily H member 2 | KCNH2 |
| MOL007206 | Mitogen-activated protein kinase 14 | MAPK14 |
| MOL007206 | Mu-type opioid receptor | MOR-1 |
| MOL007206 | Sodium-dependent noradrenaline transporter | NAT1 |
| MOL007206 | Delta-type opioid receptor | OPRD1 |
| MOL007206 | CGMP-inhibited 3',5'-cyclic phosphodiesterase A | PDE3A |
| MOL007206 | Proto-oncogene serine/threonine-protein kinase Pim-1 | PIM1 |
| MOL007206 | Peroxisome proliferator activated receptor gamma | PPARG |
| MOL007206 | mRNA of PKA Catalytic Subunit C-alpha | PRKACA |
| MOL007206 | Trypsin-1 | PRSS1 |
| MOL007206 | Prostaglandin G/H synthase 2 | PTGS2 |
| MOL007206 | Retinoic acid receptor RXR-alpha | RXRA |
| MOL007206 | Sodium channel protein type 5 subunit alpha | SCN5A |
| MOL007206 | Sodium-dependent dopamine transporter | SLC6A3 |
| MOL007206 | Sodium-dependent serotonin transporter | SLC6A4 |
| MOL007207 | Alpha-1A adrenergic receptor | ADRA1A |
| MOL007207 | Alpha-1B adrenergic receptor | ADRA1B |
| MOL007207 | Alpha-1D adrenergic receptor | ADRA1D |
| MOL007207 | Alpha-2A adrenergic receptor | ADRA2A |
| MOL007207 | Alpha-2C adrenergic receptor | ADRA2B |
| MOL007207 | Alpha-2B adrenergic receptor | ADRA2B |
| MOL007207 | Beta-1 adrenergic receptor | ADRB1R |
| MOL007207 | Beta-2 adrenergic receptor | ADRB2 |
| MOL007207 | Androgen receptor | AR |
| MOL007207 | Calmodulin | CALM1 |
| MOL007207 | Cyclin-A2 | CCNA2 |
| MOL007207 | Dipeptidyl peptidase IV | CD26 |
| MOL007207 | Cell division protein kinase 2 | Cdk2 |
| MOL007207 | Serine/threonine-protein kinase Chk1 | Chk1 |
| MOL007207 | Muscarinic acetylcholine receptor M1 | CHRM1 |
| MOL007207 | Muscarinic acetylcholine receptor M3 | Chrm3 |
| MOL007207 | Muscarinic acetylcholine receptor M4 | CHRM4 |
| MOL007207 | Prostaglandin G/H synthase 1 | COX1 |
| MOL007207 | Estrogen receptor | ESR1 |
| MOL007207 | Estrogen receptor beta | ESR2 |
| MOL007207 | Thrombin | F2 |
| MOL007207 | Glycogen synthase kinase-3 beta | GSK3B |
| MOL007207 | Heat shock protein HSP 90 | HSP90 |
| MOL007207 | Nitric oxide synthase, inducible | iNOS |
| MOL007207 | Mitogen-activated protein kinase 14 | MAPK14 |
| MOL007207 | Nuclear receptor coactivator 2 | NCOA2 |
| MOL007207 | CGMP-inhibited 3',5'-cyclic phosphodiesterase A | PDE3A |
| MOL007207 | Proto-oncogene serine/threonine-protein kinase Pim-1 | PIM1 |
| MOL007207 | Peroxisome proliferator activated receptor gamma | PPARG |
| MOL007207 | Trypsin-1 | PRSS1 |
| MOL007207 | Prostaglandin G/H synthase 2 | PTGS2 |
| MOL007207 | Retinoic acid receptor RXR-alpha | RXRA |
| MOL007207 | Sodium channel protein type 5 subunit alpha | SCN5A |
| MOL007207 | Sodium-dependent dopamine transporter | SLC6A3 |
| MOL007207 | Sodium-dependent serotonin transporter | SLC6A4 |
| MOL007210 | Acetylcholinesterase | ACHE |
| MOL007210 | Alpha-1A adrenergic receptor | ADRA1A |
| MOL007210 | Alpha-1B adrenergic receptor | ADRA1B |
| MOL007210 | Alpha-1D adrenergic receptor | ADRA1D |
| MOL007210 | Alpha-2B adrenergic receptor | ADRA2B |
| MOL007210 | Beta-2 adrenergic receptor | ADRB2 |
| MOL007210 | Androgen receptor | AR |
| MOL007210 | Cyclin-A2 | CCNA2 |
| MOL007210 | Dipeptidyl peptidase IV | CD26 |
| MOL007210 | Cell division protein kinase 2 | Cdk2 |
| MOL007210 | Serine/threonine-protein kinase Chk1 | Chk1 |
| MOL007210 | Muscarinic acetylcholine receptor M1 | CHRM1 |
| MOL007210 | Muscarinic acetylcholine receptor M3 | Chrm3 |
| MOL007210 | Muscarinic acetylcholine receptor M4 | CHRM4 |
| MOL007210 | Muscarinic acetylcholine receptor M5 | CHRM5 |
| MOL007210 | Neuronal acetylcholine receptor subunit alpha-2 | CHRNA2 |
| MOL007210 | Neuronal acetylcholine receptor protein, alpha-7 chain | CHRNA7 |
| MOL007210 | Prostaglandin G/H synthase 1 | COX1 |
| MOL007210 | Dopamine D1 receptor | DRD1 |
| MOL007210 | Estrogen receptor | ESR1 |
| MOL007210 | Estrogen receptor beta | ESR2 |
| MOL007210 | Gamma-aminobutyric acid receptor subunit alpha-1 | GABRA1 |
| MOL007210 | Heat shock protein HSP 90 | HSP90 |
| MOL007210 | 5-hydroxytryptamine 2A receptor | HTR2A |
| MOL007210 | 5-hydroxytryptamine 2C receptor | HTR2C |
| MOL007210 | 5-hydroxytryptamine receptor 3A | HTR3A |
| MOL007210 | Mu-type opioid receptor | MOR-1 |
| MOL007210 | Sodium-dependent noradrenaline transporter | NAT1 |
| MOL007210 | Delta-type opioid receptor | OPRD1 |
| MOL007210 | Proto-oncogene serine/threonine-protein kinase Pim-1 | PIM1 |
| MOL007210 | Prostaglandin G/H synthase 2 | PTGS2 |
| MOL007210 | Retinoic acid receptor RXR-alpha | RXRA |
| MOL007210 | Sodium channel protein type 5 subunit alpha | SCN5A |
| MOL007210 | Sodium-dependent dopamine transporter | SLC6A3 |
| MOL007210 | Sodium-dependent serotonin transporter | SLC6A4 |
| MOL007213 | Acetylcholinesterase | ACHE |
| MOL007213 | Alpha-1A adrenergic receptor | ADRA1A |
| MOL007213 | Alpha-1B adrenergic receptor | ADRA1B |
| MOL007213 | Alpha-1D adrenergic receptor | ADRA1D |
| MOL007213 | Alpha-2B adrenergic receptor | ADRA2B |
| MOL007213 | Beta-2 adrenergic receptor | ADRB2 |
| MOL007213 | Androgen receptor | AR |
| MOL007213 | Cyclin-A2 | CCNA2 |
| MOL007213 | Dipeptidyl peptidase IV | CD26 |
| MOL007213 | Cell division protein kinase 2 | Cdk2 |
| MOL007213 | Serine/threonine-protein kinase Chk1 | Chk1 |
| MOL007213 | Muscarinic acetylcholine receptor M1 | CHRM1 |
| MOL007213 | Muscarinic acetylcholine receptor M3 | Chrm3 |
| MOL007213 | Muscarinic acetylcholine receptor M4 | CHRM4 |
| MOL007213 | Muscarinic acetylcholine receptor M5 | CHRM5 |
| MOL007213 | Neuronal acetylcholine receptor subunit alpha-2 | CHRNA2 |
| MOL007213 | Neuronal acetylcholine receptor protein, alpha-7 chain | CHRNA7 |
| MOL007213 | Prostaglandin G/H synthase 1 | COX1 |
| MOL007213 | D(2) dopamine receptor | DRD2 |
| MOL007213 | Estrogen receptor | ESR1 |
| MOL007213 | Estrogen receptor beta | ESR2 |
| MOL007213 | Gamma-aminobutyric acid receptor subunit alpha-1 | GABRA1 |
| MOL007213 | 5-hydroxytryptamine 2A receptor | HTR2A |
| MOL007213 | 5-hydroxytryptamine 2C receptor | HTR2C |
| MOL007213 | 5-hydroxytryptamine receptor 3A | HTR3A |
| MOL007213 | Mu-type opioid receptor | MOR-1 |
| MOL007213 | Sodium-dependent noradrenaline transporter | NAT1 |
| MOL007213 | Delta-type opioid receptor | OPRD1 |
| MOL007213 | Proto-oncogene serine/threonine-protein kinase Pim-1 | PIM1 |
| MOL007213 | Prostaglandin G/H synthase 2 | PTGS2 |
| MOL007213 | Retinoic acid receptor RXR-alpha | RXRA |
| MOL007213 | Sodium channel protein type 5 subunit alpha | SCN5A |
| MOL007213 | Sodium-dependent dopamine transporter | SLC6A3 |
| MOL007213 | Sodium-dependent serotonin transporter | SLC6A4 |
| MOL007214 | Androgen receptor | AR |
| MOL007214 | Carbonic anhydrase II | CA2 |
| MOL007214 | Dipeptidyl peptidase IV | CD26 |
| MOL007214 | Cell division protein kinase 2 | Cdk2 |
| MOL007214 | Prostaglandin G/H synthase 1 | COX1 |
| MOL007214 | Estrogen receptor | ESR1 |
| MOL007214 | Estrogen receptor beta | ESR2 |
| MOL007214 | Glycogen synthase kinase-3 beta | GSK3B |
| MOL007214 | Heat shock protein HSP 90 | HSP90 |
| MOL007214 | Nitric oxide synthase, inducible | iNOS |
| MOL007214 | Beta-lactamase | LACTB |
| MOL007214 | Mitogen-activated protein kinase 14 | MAPK14 |
| MOL007214 | Proto-oncogene serine/threonine-protein kinase Pim-1 | PIM1 |
| MOL007214 | Peroxisome proliferator activated receptor gamma | PPARG |
| MOL007214 | Prostaglandin G/H synthase 2 | PTGS2 |
| MOL007214 | mRNA of Protein-tyrosine phosphatase, non-receptor type 1 | PTPN1 |
| MOL007217 | Androgen receptor | AR |
| MOL007217 | Carbonic anhydrase II | CA2 |
| MOL007217 | Cell division protein kinase 2 | Cdk2 |
| MOL007217 | Estrogen receptor | ESR1 |
| MOL007217 | Estrogen receptor beta | ESR2 |
| MOL007217 | Glycogen synthase kinase-3 beta | GSK3B |
| MOL007217 | Heat shock protein HSP 90 | HSP90 |
| MOL007217 | Nitric oxide synthase, inducible | iNOS |
| MOL007217 | Beta-lactamase | LACTB |
| MOL007217 | Mitogen-activated protein kinase 14 | MAPK14 |
| MOL007217 | Proto-oncogene serine/threonine-protein kinase Pim-1 | PIM1 |
| MOL007217 | Prostaglandin G/H synthase 2 | PTGS2 |
| MOL007217 | mRNA of Protein-tyrosine phosphatase, non-receptor type 1 | PTPN1 |
| MOL007217 | DNA topoisomerase II | TOP2 |
| MOL007218 | Acetylcholinesterase | ACHE |
| MOL007218 | Alpha-1B adrenergic receptor | ADRA1B |
| MOL007218 | Alpha-1D adrenergic receptor | ADRA1D |
| MOL007218 | Beta-2 adrenergic receptor | ADRB2 |
| MOL007218 | Androgen receptor | AR |
| MOL007218 | Dipeptidyl peptidase IV | CD26 |
| MOL007218 | Cell division protein kinase 2 | Cdk2 |
| MOL007218 | Muscarinic acetylcholine receptor M1 | CHRM1 |
| MOL007218 | Muscarinic acetylcholine receptor M3 | Chrm3 |
| MOL007218 | Muscarinic acetylcholine receptor M5 | CHRM5 |
| MOL007218 | Neuronal acetylcholine receptor subunit alpha-2 | CHRNA2 |
| MOL007218 | Neuronal acetylcholine receptor protein, alpha-7 chain | CHRNA7 |
| MOL007218 | Prostaglandin G/H synthase 1 | COX1 |
| MOL007218 | Estrogen receptor | ESR1 |
| MOL007218 | Estrogen receptor beta | ESR2 |
| MOL007218 | Thrombin | F2 |
| MOL007218 | Gamma-aminobutyric acid receptor subunit alpha-1 | GABRA1 |
| MOL007218 | 5-hydroxytryptamine receptor 3A | HTR3A |
| MOL007218 | Mu-type opioid receptor | MOR-1 |
| MOL007218 | Delta-type opioid receptor | OPRD1 |
| MOL007218 | Proto-oncogene serine/threonine-protein kinase Pim-1 | PIM1 |
| MOL007218 | Prostaglandin G/H synthase 2 | PTGS2 |
| MOL007218 | Retinoic acid receptor RXR-alpha | RXRA |
| MOL007218 | Sodium channel protein type 5 subunit alpha | SCN5A |
| MOL007218 | Sodium-dependent serotonin transporter | SLC6A4 |
| MOL007514 | Acetylcholinesterase | ACHE |
| MOL007514 | Estrogen receptor | ESR1 |
| MOL007514 | Thrombin | F2 |
| MOL007514 | Nuclear receptor coactivator 2 | NCOA2 |
| MOL007514 | Nitric-oxide synthase, endothelial | NOS |
| MOL007514 | Peroxisome proliferator activated receptor gamma | PPARG |
| MOL007879 | Acetylcholinesterase | ACHE |
| MOL007879 | Alpha-1B adrenergic receptor | ADRA1B |
| MOL007879 | Alpha-1D adrenergic receptor | ADRA1D |
| MOL007879 | Beta-2 adrenergic receptor | ADRB2 |
| MOL007879 | Androgen receptor | AR |
| MOL007879 | Beta-secretase | BACE |
| MOL007879 | Carbonic anhydrase II | CA2 |
| MOL007879 | Calmodulin | CALM1 |
| MOL007879 | Cyclin-A2 | CCNA2 |
| MOL007879 | Dipeptidyl peptidase IV | CD26 |
| MOL007879 | Cell division protein kinase 2 | Cdk2 |
| MOL007879 | Serine/threonine-protein kinase Chk1 | Chk1 |
| MOL007879 | Prostaglandin G/H synthase 1 | COX1 |
| MOL007879 | Estrogen receptor | ESR1 |
| MOL007879 | Estrogen receptor beta | ESR2 |
| MOL007879 | Coagulation factor Xa | F10 |
| MOL007879 | Thrombin | F2 |
| MOL007879 | Coagulation factor VII | F7 |
| MOL007879 | Glycogen synthase kinase-3 beta | GSK3B |
| MOL007879 | Heat shock protein HSP 90 | HSP90 |
| MOL007879 | Nitric oxide synthase, inducible | iNOS |
| MOL007879 | Potassium voltage-gated channel subfamily H member 2 | KCNH2 |
| MOL007879 | Calcium-activated potassium channel subunit alpha 1 | KCNMA1 |
| MOL007879 | Mitogen-activated protein kinase 14 | MAPK14 |
| MOL007879 | Nuclear receptor coactivator 1 | NCOA1 |
| MOL007879 | Nuclear receptor coactivator 2 | NCOA2 |
| MOL007879 | Nitric-oxide synthase, endothelial | NOS |
| MOL007879 | Proto-oncogene serine/threonine-protein kinase Pim-1 | PIM1 |
| MOL007879 | Peroxisome proliferator activated receptor gamma | PPARG |
| MOL007879 | mRNA of PKA Catalytic Subunit C-alpha | PRKACA |
| MOL007879 | Trypsin-1 | PRSS1 |
| MOL007879 | Prostaglandin G/H synthase 2 | PTGS2 |
| MOL007879 | mRNA of Protein-tyrosine phosphatase, non-receptor type 1 | PTPN1 |
| MOL007879 | Pregnane X receptor | PXR |
| MOL007879 | Retinoic acid receptor RXR-alpha | RXRA |
| MOL007879 | Sodium channel protein type 5 subunit alpha | SCN5A |
| MOL007879 | DNA topoisomerase II | TOP2 |
| MOL008121 | Acetylcholinesterase | ACHE |
| MOL008121 | Thrombin | F2 |
| MOL008121 | Nuclear receptor coactivator 2 | NCOA2 |
| MOL008121 | Nitric-oxide synthase, endothelial | NOS |
| MOL008121 | Peroxisome proliferator activated receptor gamma | PPARG |
| MOL008391 | Androgen receptor | AR |
| MOL008391 | Estrogen receptor | ESR1 |
| MOL008393 | Tubulin beta-1 chain | TUBB |
| MOL008397 | Androgen receptor | AR |
| MOL008397 | Estrogen receptor | ESR1 |
| MOL008397 | Glucocorticoid receptor | NR3C1 |
| MOL008400 | Amyloid beta A4 protein | APP |
| MOL008400 | Androgen receptor | AR |
| MOL008400 | Carbonic anhydrase II | CA2 |
| MOL008400 | Calmodulin | CALM1 |
| MOL008400 | Cyclin-A2 | CCNA2 |
| MOL008400 | Cell division protein kinase 2 | Cdk2 |
| MOL008400 | Serine/threonine-protein kinase Chk1 | Chk1 |
| MOL008400 | Prostaglandin G/H synthase 1 | COX1 |
| MOL008400 | Estrogen receptor | ESR1 |
| MOL008400 | Estrogen receptor beta | ESR2 |
| MOL008400 | Glycogen synthase kinase-3 beta | GSK3B |
| MOL008400 | Heat shock protein HSP 90 | HSP90 |
| MOL008400 | Nitric oxide synthase, inducible | iNOS |
| MOL008400 | Mitogen-activated protein kinase 14 | MAPK14 |
| MOL008400 | Collagenase 3 | MMP13 |
| MOL008400 | Neutrophil collagenase | MMP8 |
| MOL008400 | Nuclear receptor coactivator 1 | NCOA1 |
| MOL008400 | CGMP-inhibited 3',5'-cyclic phosphodiesterase A | PDE3A |
| MOL008400 | Proto-oncogene serine/threonine-protein kinase Pim-1 | PIM1 |
| MOL008400 | Peroxisome proliferator activated receptor gamma | PPARG |
| MOL008400 | mRNA of PKA Catalytic Subunit C-alpha | PRKACA |
| MOL008400 | Trypsin-1 | PRSS1 |
| MOL008400 | Prostaglandin G/H synthase 2 | PTGS2 |
| MOL008400 | mRNA of Protein-tyrosine phosphatase, non-receptor type 1 | PTPN1 |
| MOL008400 | Retinoic acid receptor RXR-alpha | RXRA |
| MOL008407 | Androgen receptor | AR |
| MOL008407 | Estrogen receptor | ESR1 |
| MOL008407 | Glucocorticoid receptor | NR3C1 |
| MOL008407 | Mineralocorticoid receptor | NR3C2 |
| MOL008407 | Progesterone receptor | PGR |
| MOL008411 | Cell division protein kinase 2 | Cdk2 |
| MOL008411 | Estrogen receptor | ESR1 |
| MOL008411 | Mu-type opioid receptor | MOR-1 |
| MOL008411 | Sodium channel protein type 5 subunit alpha | SCN5A |
| MOL013277 | Acetylcholinesterase | ACHE |
| MOL013277 | Beta-2 adrenergic receptor | ADRB2 |
| MOL013277 | Androgen receptor | AR |
| MOL013277 | Carbonic anhydrase II | CA2 |
| MOL013277 | Calmodulin | CALM1 |
| MOL013277 | Dipeptidyl peptidase IV | CD26 |
| MOL013277 | Serine/threonine-protein kinase Chk1 | Chk1 |
| MOL013277 | Prostaglandin G/H synthase 1 | COX1 |
| MOL013277 | Estrogen receptor | ESR1 |
| MOL013277 | Estrogen receptor beta | ESR2 |
| MOL013277 | Coagulation factor Xa | F10 |
| MOL013277 | Thrombin | F2 |
| MOL013277 | Coagulation factor VII | F7 |
| MOL013277 | Heat shock protein HSP 90 | HSP90 |
| MOL013277 | Nitric oxide synthase, inducible | iNOS |
| MOL013277 | Potassium voltage-gated channel subfamily H member 2 | KCNH2 |
| MOL013277 | Calcium-activated potassium channel subunit alpha 1 | KCNMA1 |
| MOL013277 | Nuclear receptor coactivator 1 | NCOA1 |
| MOL013277 | Nuclear receptor coactivator 2 | NCOA2 |
| MOL013277 | Nitric-oxide synthase, endothelial | NOS |
| MOL013277 | Proto-oncogene serine/threonine-protein kinase Pim-1 | PIM1 |
| MOL013277 | Peroxisome proliferator activated receptor gamma | PPARG |
| MOL013277 | Trypsin-1 | PRSS1 |
| MOL013277 | Prostaglandin G/H synthase 2 | PTGS2 |
| MOL013277 | mRNA of Protein-tyrosine phosphatase, non-receptor type 1 | PTPN1 |
| MOL013277 | Sodium channel protein type 5 subunit alpha | SCN5A |
| MOL013277 | Sulfonylurea receptor 1 | SUR1 |
| MOL013277 | DNA topoisomerase II | TOP2 |
| MOL013279 | Alpha-1B adrenergic receptor | ADRA1B |
| MOL013279 | Alpha-2C adrenergic receptor | ADRA2B |
| MOL013279 | Beta-2 adrenergic receptor | ADRB2 |
| MOL013279 | Androgen receptor | AR |
| MOL013279 | Carbonic anhydrase II | CA2 |
| MOL013279 | Calmodulin | CALM1 |
| MOL013279 | Cyclin-A2 | CCNA2 |
| MOL013279 | Dipeptidyl peptidase IV | CD26 |
| MOL013279 | Cell division protein kinase 2 | Cdk2 |
| MOL013279 | Serine/threonine-protein kinase Chk1 | Chk1 |
| MOL013279 | Prostaglandin G/H synthase 1 | COX1 |
| MOL013279 | Estrogen receptor | ESR1 |
| MOL013279 | Estrogen receptor beta | ESR2 |
| MOL013279 | Coagulation factor Xa | F10 |
| MOL013279 | Glycogen synthase kinase-3 beta | GSK3B |
| MOL013279 | Heat shock protein HSP 90 | HSP90 |
| MOL013279 | Nitric oxide synthase, inducible | iNOS |
| MOL013279 | Mitogen-activated protein kinase 14 | MAPK14 |
| MOL013279 | Nuclear receptor coactivator 2 | NCOA2 |
| MOL013279 | Nitric-oxide synthase, endothelial | NOS |
| MOL013279 | Proto-oncogene serine/threonine-protein kinase Pim-1 | PIM1 |
| MOL013279 | Peroxisome proliferator activated receptor gamma | PPARG |
| MOL013279 | Trypsin-1 | PRSS1 |
| MOL013279 | Prostaglandin G/H synthase 2 | PTGS2 |
| MOL013279 | mRNA of Protein-tyrosine phosphatase, non-receptor type 1 | PTPN1 |
| MOL013279 | Sodium channel protein type 5 subunit alpha | SCN5A |
| MOL013428 | DNA topoisomerase II | TOP2 |
| MOL013430 | Androgen receptor | AR |
| MOL013430 | Dipeptidyl peptidase IV | CD26 |
| MOL013430 | Cell division protein kinase 2 | Cdk2 |
| MOL013430 | Estrogen receptor | ESR1 |
| MOL013430 | Thrombin | F2 |
| MOL013430 | Glycogen synthase kinase-3 beta | GSK3B |
| MOL013430 | Nitric oxide synthase, inducible | iNOS |
| MOL013430 | Mitogen-activated protein kinase 14 | MAPK14 |
| MOL013430 | Prostaglandin G/H synthase 2 | PTGS2 |
| MOL013435 | Androgen receptor | AR |
| MOL013435 | Estrogen receptor | ESR1 |
| MOL013435 | Thrombin | F2 |
| MOL013435 | Glycogen synthase kinase-3 beta | GSK3B |
| MOL013435 | Nitric oxide synthase, inducible | iNOS |
| MOL013435 | Prostaglandin G/H synthase 2 | PTGS2 |
| MOL013436 | Beta-2 adrenergic receptor | ADRB2 |
| MOL013436 | Androgen receptor | AR |
| MOL013436 | Cell division protein kinase 2 | Cdk2 |
| MOL013436 | Estrogen receptor | ESR1 |
| MOL013436 | Thrombin | F2 |
| MOL013436 | Glycogen synthase kinase-3 beta | GSK3B |
| MOL013436 | Nitric oxide synthase, inducible | iNOS |
| MOL013436 | Mitogen-activated protein kinase 14 | MAPK14 |
| MOL013436 | CGMP-inhibited 3',5'-cyclic phosphodiesterase A | PDE3A |
| MOL013436 | Prostaglandin G/H synthase 2 | PTGS2 |
| MOL013437 | Alpha-1B adrenergic receptor | ADRA1B |
| MOL013437 | Beta-2 adrenergic receptor | ADRB2 |
| MOL013437 | Androgen receptor | AR |
| MOL013437 | Carbonic anhydrase II | CA2 |
| MOL013437 | Calmodulin | CALM1 |
| MOL013437 | Dipeptidyl peptidase IV | CD26 |
| MOL013437 | Muscarinic acetylcholine receptor M1 | CHRM1 |
| MOL013437 | Muscarinic acetylcholine receptor M3 | Chrm3 |
| MOL013437 | Estrogen receptor | ESR1 |
| MOL013437 | Estrogen receptor beta | ESR2 |
| MOL013437 | Coagulation factor Xa | F10 |
| MOL013437 | Thrombin | F2 |
| MOL013437 | Nitric oxide synthase, inducible | iNOS |
| MOL013437 | Nuclear receptor coactivator 2 | NCOA2 |
| MOL013437 | Nitric-oxide synthase, endothelial | NOS |
| MOL013437 | CGMP-inhibited 3',5'-cyclic phosphodiesterase A | PDE3A |
| MOL013437 | Peroxisome proliferator activated receptor gamma | PPARG |
| MOL013437 | Trypsin-1 | PRSS1 |
| MOL013437 | Prostaglandin G/H synthase 2 | PTGS2 |
| MOL013437 | Retinoic acid receptor RXR-alpha | RXRA |
| MOL013440 | Thrombin | F2 |
