# Supplementary figures and images for: Study on the Effect and Mechanism of Huaji Jianpi Decoction on Simple Obesity
Source: Evid Based Complement Alternat Med. 2022 Apr 28;2022:5494224. doi: 10.1155/2022/5494224 (PMC9071864; doi:10.1155/2022/5494224)

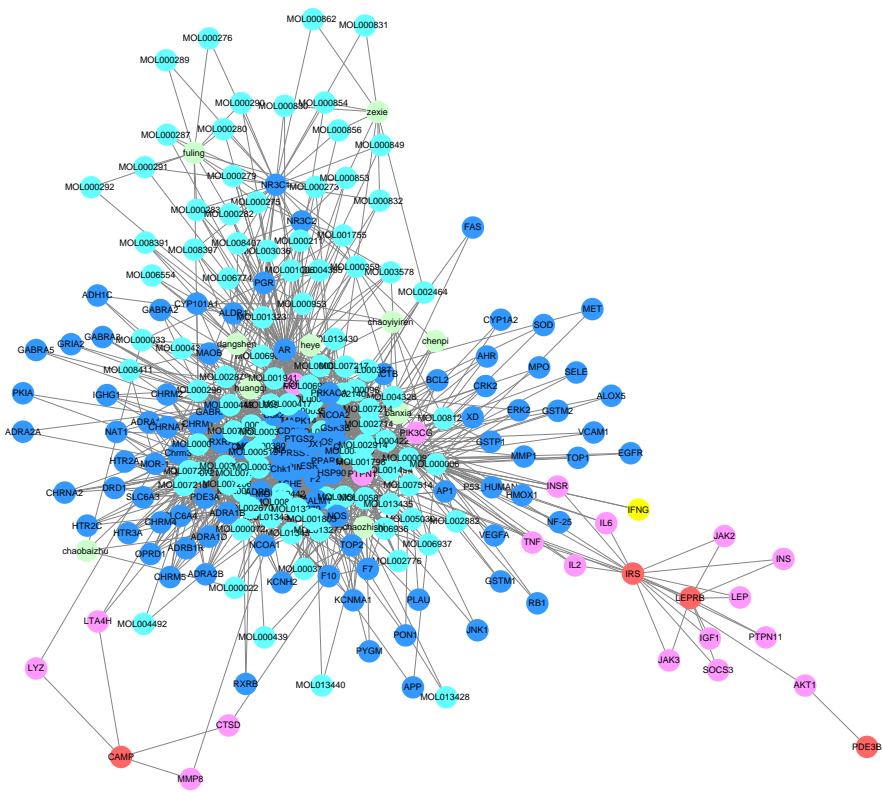

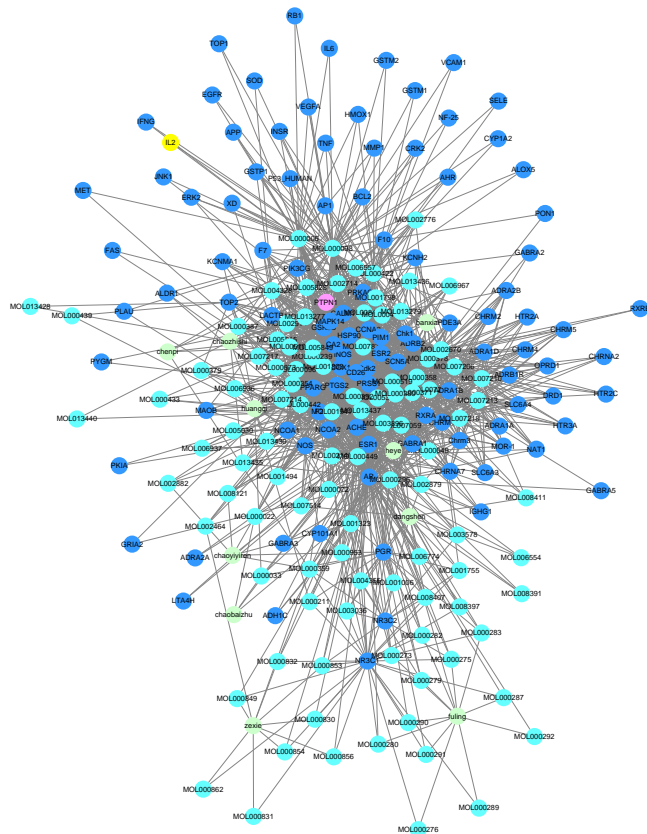

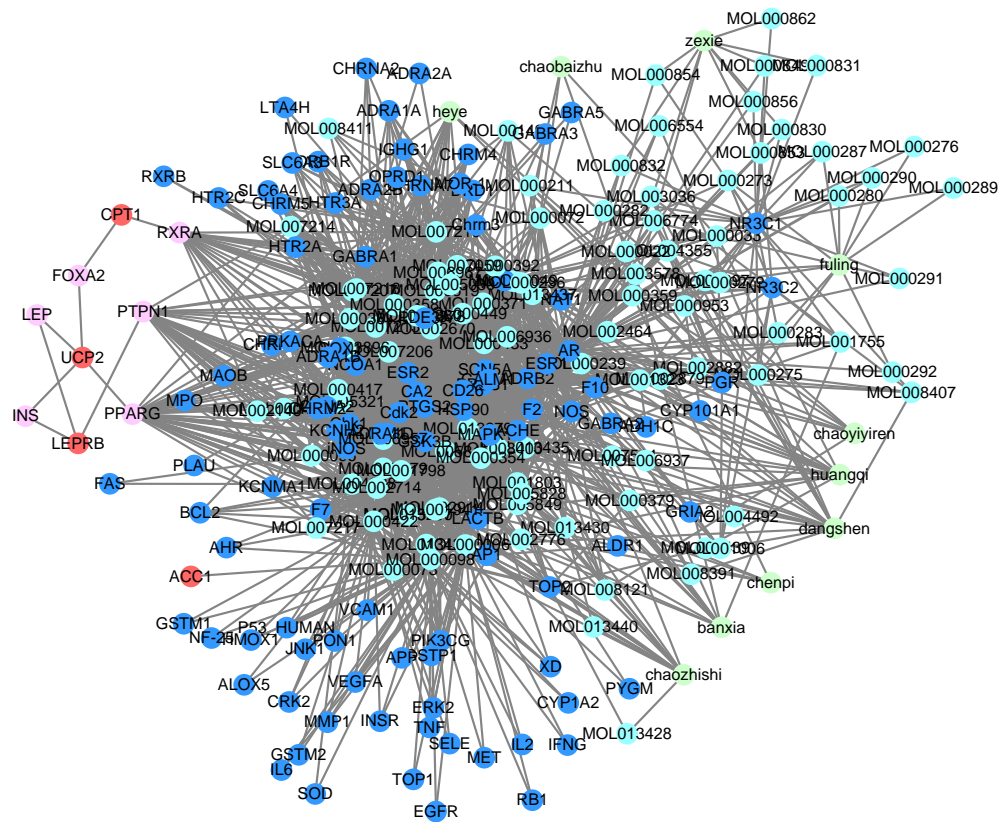

Supplement: Supplementary Materials — Supplementary Table S1: The information of all components of HJJPD. Supplementary Table S2: Targets of 169 components. Supplementary S3: The biological network of HJJPD acts on the proteins related to the AMPK-ACC pathway, LepRb-IRS-PI3K-PDE3B-cAMP pathway, and LepRb-SHP2-MAPKs (ERK1/2) pathway. [file 5494224.f1.zip › 5494224.f1/Supplementary S3.pdf]
